# Protocol to develop and pilot a primary mental healthcare intervention model to address the medium- to long-term Ebola associated psychological distress and psychosocial problems in Mubende District in Central Uganda (the Ebola+D project)

**DOI:** 10.1371/journal.pone.0329591

**Published:** 2025-08-06

**Authors:** Leticia Kyohangirwe, Richard S. Mpango, Christine Tusiime, Rwamahe Rutakumwa, Joshua Ssebunnya, Andrew Obuku, Barbra Elsa Kiconco, Isaac Sekitoleko, Hafsa Sentongo, Kenneth Kalani, Wilson Muhwezi, Giulia Greco, Ricardo Araya Baltra, Birthe Loa Knizek, Pontiano Kaleebu, Valeria Mondelli, Nambusi Kyegombe, Patrick Tenywa, Philip Amanyire, Wilber Ssembajjwe, Crick Lund, Eugene Kinyanda

**Affiliations:** 1 Mental Health Focus Area of the Medical Research Council, Uganda Virus Research Institute & London School of Hygiene and Tropical Medicine, Uganda Research Unit, Uganda; 2 Butabika National Referral Mental Hospital, Kampala, Uganda; 3 Brown School, Washington University in St. Louis, St. Louis, Missouri, United States of America; 4 Department of Mental Health and Control of Substance Use, Ministry of Health, Uganda; 5 Department of Psychiatry, Makerere University College of Health Sciences, Kampala, Uganda; 6 Centre for Health Economics, London School of Hygiene & Tropical Medicine, London, United Kingdom; 7 Centre for Global Mental Health, Health Service and Population Research Department, Institute of Psychiatry, Psychology and Neuroscience, Kings College London, London, United Kingdom; 8 Department of Mental Health, Norwegian University of Science and Technology, Norway; 9 Basic Sciences, Uganda Medical Research Council/Uganda Virus Research Institute & London School of Hygiene and Tropical Medicine, Uganda Research Unit, Uganda; 10 Maurice Wohl Clinical Neuroscience Institute, Kings College London, London, United Kingdom; 11 Social Science, Medical Research Council/Uganda Virus Research Institute & London School of Hygiene and Tropical Medicine, Uganda Research Unit, Uganda; 12 Alan J Flisher Centre for Public Mental Health, Department of Psychiatry and Mental Health, University of Cape Town, South Africa; Regional Labor Institute, Directorate General Factory Advice Service and Labour Institutes (DGFASLI), Ministry of Labour and Employment, Government of India, INDIA

## Abstract

Ebola Virus Disease (EVD) presents significant global health challenges, including high mortality and substantial physical morbidity among patients and survivors. Beyond immediate health impacts, EVD survivors, frontline healthcare workers, and community members face profound mental health and psychosocial issues. Over 35 EVD outbreaks have occurred in Africa since 1976, often in the context of fragile health systems and chronic conflict, complicating the response to mental health needs. Uganda has experienced seven outbreaks, the latest from September 20, 2022, to January 11, 2023, affecting nine districts, with Mubende as the epicenter. The Mental Health Focus Area of the Medical Research Council/Uganda Virus Research Institute and London School of Hygiene and Tropical Medicine, Uganda Research Unit, in collaboration with Uganda’s Ministry of Health, has initiated the development and piloting of the Ebola+D mental health intervention to address the medium- and long-term mental health consequences of Ebola in the Mubende district. This intervention will be a collaborative stepped care model based on the successful HIV + D intervention in Uganda and the MANAS intervention in India. Participatory, theory-informed approaches will be employed in Mubende district to develop the Ebola+D mental health intervention. This will involve five phases: i) adaptation of the HIV + D collaborative stepped care mental health intervention into primary health care in Mubende district to produce the Ebola+D mental health intervention; ii) adaptation and translation of the Problem Solving Therapy for Primary Care (PST-PC) treatment manual to the local rural situation in Mubende district; iii) a pilot study to evaluate the acceptability, feasibility and impact of the Ebola+D mental health intervention on mental health outcomes; iv) a health economics component to examine the costs of the Ebola + D mental health intervention; and v) a qualitative component to explore the Ebola virus disease (EVD) associated negative beliefs and lived out experiences of affected members of the community. The findings from this study will inform future mental health and psychosocial interventions secondary to outbreaks of Emerging Viral Diseases (such as EVD) in low resourced settings such those in sub-Saharan Africa.

**Trial registration:**

ClinicalTrials.gov NCT06093646

## Introduction

In 2014, the World Health Organisation (WHO) declared the 2014–2016 West African Ebola outbreak as a “public health emergency of international concern”, an epidemic that eventually engulfed three West African countries of Guinea, Sierra Leone, and Liberia, infected 28,616 persons, led to 11,310 deaths and left over 10,000 survivors [1]. Ebola Virus Disease (EVD) is now considered a threat to public health in the heavily populated regions of east, central and west Africa where since 1976 at least 35 EVD outbreaks have been reported [2]. To-date, Uganda has had 7 Ebola outbreaks with the most recent having occurred between 20^th^ September 2022 to the 11^th^ of January 2023, affected 9 districts (Mubende district was the epicentre), infected 164 persons (142 confirmed and 22 probable), led to 55 confirmed deaths, with 87 survivors and 4,000 contacts [3].

EVD is not only associated with a high mortality and physical morbidity, but also with mental health disorders. EVD related mental health disorders do not only affect EVD survivors, but also frontline health workers and volunteers and members of the affected communities [2,4–8]. Ebola survivors have been reported to develop the mental health problems of depression, anxiety, post-traumatic stress disorder (PTSD) and insomnia. These occur as a result of a number of factors including the trauma associated with the EVD illness experience (including the manner of evacuation from home that involves destruction of personal effects to prevent transmission of disease, and the harrowing experience in the Ebola treatment unit), being infected with a highly fatal disease, loss of loved ones, rejection, isolation stigmatisation by the community, and possibly as a result of secondary central nervous system (CNS) viral invasion with recent evidence pointing towards clinical and imaging features indicative of meningoencephalitis and meningitis [2,4–8]. For healthcare workers, providing medical care for EVD infected patients is mentally very stressful due to treating very sick patients, the high mortality in the Ebola treatment units, extended work shift times, risk of infection, fear of infecting family members, witnessing the death of colleagues, and having to wear personal protective equipment (which is uncomfortable, impairs communication and performance of diagnostic and therapeutic procedures). These all contribute to predisposing frontline health workers and volunteers to anxiety, depression, PTSD, fatigue and social isolation [9,10]. For members of Ebola affected communities, loss of loved ones, having loved ones isolated in Ebola treatment units and isolation centres, rejection and stigmatisation, threatened violence and sometimes actual violence and economic and social disruption associated with community lockdowns all contribute to mental health disorders [5,10,11]. EVD associated mental health disorders if not treated have been reported to have a tendency to chronicity with Kelly et al., (2019) in a follow-up study of EVD survivors of the 1995 Ebola Outbreak in Kikwit, DRC reporting persistence of depressive and anxiety symptoms two decades after the outbreak [2].

Despite this demonstrated need, the implementation of community mental health services in EVD affected communities remains poor [12]. This has been attributed to a number of reasons that include: affected communities are often remote and suffer from chronic war conflict; health systems in many sub-Saharan African countries are fragile as demonstrated during the 2014–2016 West African Ebola outbreak where a 2015 World Bank report noted that at the time of the West African Ebola outbreak, the number of mental health workers (including psychiatrist) in the local population was as low as 1 in 6 million in Sierra Leone and 1 in 25,000 in Liberia [13]; a lack of prioritisation of mental health by international humanitarian donor agencies in preference for biomedical interventions [14,15]. Therefore, there is an urgent need to commit more resources to mental health care in vulnerable regions including developing novel approaches to addressing this mental health burden.

One endeavour in this direction is being undertaken by the Mental Health Focus Area of the Medical Research Council/Uganda Virus Research Institute and London School of Hygiene and Tropical Medicine, Uganda Research Unit (MRC/UVRI & LSHTM Uganda Research Unit) in partnership with the Ministry of Health of Uganda, entitled, *‘Proposal to address the medium- to long-term EBOLA associated psychological Distress and psychosocial problems in Mubende District in central Uganda (Ebola+D Project)’. The Ebola+D Study will* be undertaken in Mubende District in central Uganda (the epicentre of recent Uganda EVD outbreak). This protocol describes the process that will be employed to develop, and pilot test the Ebola+D mental health intervention model to address the mental health problems of the Ebola affected Mubende district.

### Mental health care in the Mubende district at the time of the 2022–2023 Ebola outbreak

Mubende district (population 720,000, with 42 public health care facilities; 1 regional referral hospital, 12 health centre III facilities and 29 health centre II facilities [16]) at the time of the Ebola outbreak had limited mental health services that primarily consisted of 1 mental health department at Mubende Regional Referral Hospital. The entire district mental health work force consisted of only 12 mental health workers (1 psychiatrist, 2 psychiatric clinical officers and 9 psychiatric nurses) all based at the regional referral hospital with no primary mental health services at the more than 42 public health facilities in the district (*Dr Kenneth Kalani, field coordinator of the Ebola MHPSS response- personal communication December 2022*). Following the Ebola outbreak in Mubende district on the 20^th^ September 2022, the Ministry of Health of Uganda, established an Ebola National Task Force that had as one of its sub-pillars the Ebola Mental Health and Psychosocial Support Services (MHPSS). The MHPSS sub-pillar outlined three objectives: i) enhance the capacity of health workers and community volunteers to offer mental health and psychosocial support, ii) extend mental health and psychosocial support services to various segments of the community including EVD survivors and members of the affected community, and iii) provide mental health and psychosocial support services specifically tailored for frontline health workers. In the immediate aftermath of the outbreak, Mubende district saw an influx of psychosocial workers who supported the district to address the immediate mental health and psychosocial effects of EVD. In preparation for the scale down of MHPSS, the Ministry of Health with support of partners trained 90 members of the Village Health Team (VHT; the first level of primary health care in the Uganda health system; 10 VHTs per sub-county) to provide continued mental health and psychosocial support to the district. However, no mechanism was put in place to provide continued support for the trained VHTs and for upward linkage of referrals to the Mental Health Department at Mubende Regional Referral Hospital.

### Ebola+D mental health intervention

To address the above identified gap, the Mental Health Focus Area of the MRC/UVRI & LSHTM Uganda Research Unit proposes to develop and pilot the Ebola+D mental health intervention model that would employ a collaborative stepped care approach modelled on the HIV + D mental health intervention (a depression integration model that has shown effectiveness against depression and anxiety disorder in adult HIV care in Uganda) [17] which was developed by the Mental Health Focus Area modelled on the MANAS mental health intervention that demonstrated effectiveness and cost-effectiveness in primary healthcare in India [18]. The development of the Ebola +D intervention will be guided by four principles: i) the intervention should address the health system challenges in the district (low mental health literacy in the community, and inadequate knowledge and skills of non-specialist primary health care workers to identify and support patients with mental health issues; severe shortage of mental health workers in the district; shortage of other cadres of health care workers; a poor referral system in the district) [19,20]; ii) use the best global practices hence the task-shifting approach of using supervised trained lay health workers to deliver low intensity psychological treatments [18,21,22]; iii) the selected clinical treatments should have been shown to be effective against the target mental disorders (depression, anxiety disorders and PTSD), hence the selection of the psychoeducation [23], the transdiagnostic Problem Solving Therapy (PST) [24], and the use of Selective-Serotonin Re-uptake Inhibitors (SSRIs) for depression, anxiety disorder and PTSD [25–27]), iv) and the intervention should be guided by the specific needs of the patient and aligned with the concepts of person-centred care [28]. The overall goal of the Ebola+D intervention will be recovery from the mental health disorders (depression, anxiety and PTSD). This will be guided by two rules: allocation of the clinical treatments based on decision rules defined by severity of symptoms and response; and planned reviews of response at regular intervals (monthly until remission). The locally validated Luganda (Luganda is the predominant local language spoken in central Uganda) or English version of the WHO- Self Report Questionnaire (SRQ-20) [29,30] will be used by the trained lay health workers (members of the Village Health Team) to screen members of the community for psychological distress. The SRQ-20 which was developed by the WHO specifically for low- and middle- income (LMIC) settings employs a yes/no answer format (which is amicable to lay health workers who often have low levels of literary) and is designed to detect non-specific psychological distress, including suicidality [29].

The Ebola +D mental health intervention will be implemented at 11 public health care facilities (health centre IIIs and IVs) in Mubende District. The intervention will be delivered by the health centre based medical team, in collaboration with members of the village health team (VHT), who constitutethe first level of the Ugandan health care system. At each facility, the intervention will be coordinated by a designated mental health contact person,either a general nurse or general clinician based at the health facility, and will be supported by mental health professionals from Mubende Regional Referral Hospital. The delivery team at each site will comprise two trained lay health workers (selected from the VHT), a supervisor (selected from the health centre staff), two clinicians from the health centre, and a visiting specialist mental health worker (psychiatric clinical officer or psychiatric nurse) affiliated with Mubende Regional Referral Hospital.

A health centre III is a key part of Uganda’s decentralised healthcare system, providing outpatient, maternal and child health, inpatient care, laboratory, and HIV/AIDS services at the sub-county level. Staffed by clinical officers, nurses, midwives, and lab technicians, it features a maternity ward, inpatient ward, outpatient department and a laboratory. It serves as a referral point for health centre II facilities and collaborates with Village Health Teams to promote public health and preventive care. A health centre IV in Uganda is a higher-level facility within decentralised health care system, serving as the sub-district’s main health unit. It provides comprehensive outpatient and inpatient care, including advanced maternal and child health services, emergency obstetric care, and minor surgical procedures. It acts as a referral point for health centre III facilities. It is staffed by a medical officer, clinical officers, nurses, midwives, laboratory technicians, and support staff.

### The Ebola ±D mental health intervention will involve 4 steps

Step 1 (Initiation of treatment): Patients with SRQ-20 scores of ≥6) (demonstrated 84% sensitivity and 93% specificity) [29] are advised about their scores and offered Psychoeducation (undertaken by a lay health worker, member of the VHT).

Step 2: (Management of moderate to severe cases): Patients who remain symptomatic at follow up (SRQ-20 score ≥6, after 4 weeks) despite Step 1. These will be offered Problem Solving Therapy (PST; minimum 4 sessions, maximum 8 sessions) (undertaken by a lay health worker, member of the VHT).

Step 3: (Monitoring outcomes): If after 6 sessions of PST, SRQ-20 scores are still 6 and above, complete PST sessions and add Selective Serotonin Re-Uptake Inhibitor (SSRI; such as Fluoxetine 20 mg/day for 6 months) (SSRI medication initiated by clinician).

Step 4: (Referral to Mental Health Specialist/ Clinician in charge of facility): If despite Step 3 there is no improvement (SRQ-20 scores 6 and above); or at initiation of treatment or during any phase of treatment, if someone is deemed to have a high suicide risk confirmed by the supervisor (contact general nurse or clinician), following a positive SRQ-20 item 16 (‘do you feel that you are a worthless person?’) or/and item 17 (‘has the thought of ending your life been in your mind?’) [29], then continue all existing treatment and refer to a specialised mental health worker (psychiatrist, psychiatric nurse or psychiatric clinical officer at Mubende Regional Referral Hospital).

To develop the Ebola+D mental health intervention, we shall employ the methodology developed by PREMIUM (Program for Effective Mental Health Interventions in Under-Resourced Health Systems) [31] where participatory, theory-informed approaches will be used. The Mental Health Focus Area of the MRC/UVRI & LSHTM Uganda Research Unit has previously employed this methodology to support the development of the HIV + D mental health intervention. This will involve the following: i) develop a Health Talk about the psychosocial and mental health problems associated with EVD to be delivered at the triage area where community members who have come to access health services at the public health care facilities are waiting (to increase mental health literacy, messages will be drawn from the WHO Psychological First Aid Manual) [32]; ii) will develop the message that will be given in the Psychoeducation Session (1^st^ step of care in the Ebola+D mental health intervention); iii) will undertake the local adaptation and translation of Problem Solving Therapy for Primary Care (PST-PC) treatment manual [33]; iv) will undertake the training and supervision of lay health workers and their supervisors; v) will undertake the training of clinicians in mhGAP guidelines (including use of SSRIs) [34].

### Steps in the development of the Ebola+D mental health intervention

The development of the Ebola+D mental health intervention was guided by prior experience of the Mental Health Research Team (which included psychologists, psychiatrists, social workers and health economists) who had participated in the HIV + D trial and in the initial psychosocial response to the Ebola epidermic in Mubende district. Through a series of meetings by the Mental Health Research team a number of factors were noted, namely: i) the lack of primary mental health services in the target district; ii) the severe shortage of mental health workers in the district; iii) the severe shortage of medical personnel in the primary health facilities; iv) the fact that the community was struggling to cope with the negative consequences of the Ebola epidermic which occurred right on the heels of the COVID-19 pandemic, the team thought that the community would benefit from problem solving training rather than behavioural activation which had earlier been successfully used in the HIV + D trial; and v) the fact that the HIV + D stepped care collaborative model had been able to successfully circumvent the above challenges in HIV care. We therefore opted to adapt the HIV + D mental health intervention into the local situation in Mubende district through the following five phases: i) adapt the HIV + D collaborative stepped care mental health intervention into primary health care in Mubende district to produce the Ebola+D mental health intervention; ii) to adapt and translate the Problem Solving Therapy for Primary Care (PST-PC) treatment manual [33] to the local rural situation in Mubende district; iii) a pilot study to evaluate the acceptability, feasibility and impact on mental health outcomes of the Ebola+D mental health intervention; iv) a health economics component to examine the costs of the Ebola + D mental health intervention from the providers’ perspective to determine how much it will cost for the health system to set up and operate the intervention at the health facility level.; and v) to explore the Ebola virus disease (EVD) associated negative beliefs and lived out experiences of affected members of the community.

Problem Solving therapy (PST) is a low intensity cognitive-behavioral intervention that focuses on training in adaptive problem-solving attitudes and skills [33]. A meta-analysis by Bell and D’Zurilla (2009) using controlled outcome studies on efficacy of PST for reducing depressive symptomatology found that PST was equally effective as other psychosocial therapies and medication treatments and significantly more effective than no treatment [35]. Zhang and colleagues (2018) in a systematic review and meta-analysis of clinical trials examining PST for patients with depression and/or anxiety in primary care reported PST’s effectiveness for primary care depression and/or anxiety [36]. Connolly and colleagues (2021) in a systematic review and meta-analysis to investigate the effectiveness of community-based mental health interventions by professionally trained, lay counsellors in low- and middle-income countries, observed that the use of professionally trained, lay counsellors to provide mental health interventions in low- and middle-income countries was associated with significant improvements in mental health symptoms across a range of settings [37]. In this project we shall adapt the Problem-Solving Therapy for Primary Care (PST-PC) treatment manual by Hegel and Areán (2011) for use to train lay health workers (members of the Village Health Team) in the rural situation of Mubende.

## Materials and methods

### Setting and design

A participatory, mixed-method study will be employed to achieve the study phases. The study will be conducted in Mubende district in central region of Uganda, 160 kilometres west of Kampala, the capital city of Uganda. The health system where the Ebola+D mental health intervention will be developed consists of a Mental Health Department at Mubende Regional Referral Hospital at the apex of a network of 42 public health facilities (1 regional referral hospital, 12 health centre IIIs and 29 health centre IIs) that primarily do not offer any mental health services apart from the regional referral hospital.

### Participants and procedure

#### 1) To adapt the HIV + D collaborative stepped care mental health intervention to produce the Ebola+D mental health intervention.

We shall employ the Theory of Change (ToC) based approach by De Silva and colleagues (2014) [38] to adapt the HIV + D mental health intervention to the post-Ebola primary health care situation in Mubende district in Uganda. This participatory methodology has previously been used in the Ugandan setting by this research team [39] and will be used to achieve the following: i) describe the proposed causal pathway to impact; ii) increase the chances of success due to its sensitivity to the local context, stakeholder involvement and hence promotion of stakeholder buy-in; iii) help identify barriers and strategies needed to implement the HIV + D mental health intervention in post-Ebola Mubende district so as to ensure full integration and the achievement of the ultimate goal of recovery from Ebola associated mental health problems (depression, anxiety and Post-traumatic stress disorder); and iv) identify possible outcome indicators of the intervention.

Iterative theory of change (ToC) workshops will be held with the different categories of stakeholders who can influence the implementation of the intervention. Through a process of participatory discussions, consensus will be sought on the programme’s desired impact of having the integration of the management of common mental health problems in public health care facilities in Mubende district, Uganda. Each group will develop a programme theory describing how the intervention is expected to unfold and attain the desired impact. Also, to be discussed are the different cadres who will deliver and support the different steps of care in the Ebola+D mental health integration model, how these will be implemented, the competencies required to perform these roles and responsibilities and how these will be acquired and maintained, and the monitoring of quality and fidelity of treatment.

A final workshop, bringing together all willing stakeholders will be conducted. Findings from the separate ToC workshops will be presented, and consensus will be made on the causal pathway to impact, the intervention and indicators, assumptions, and rationale for each point along the causal pathway, a ToC map will be developed.

#### 2) To adapt and translate the Problem Solving Therapy for Primary Care (PST-PC) treatment manual to the local rural situation in Mubende district.

To locally adapt the problem solving therapy for primary care (PST-PC) manual to the post-Ebola situation in rural Mubende district, we shall employ a methodology similar to that used by Chowdhary and others, (2016) [31] in India and most recently used by Kinyanda and colleagues (2020) [17] to adapt Behavioural Activation therapy to the local HIV care situation in Uganda.

The adaptation process will involve presenting the PST-PC manual to different categories of stakeholders at separate workshops. The first workshop with mental health professionals (psychiatrists, psychologists, social workers and psychiatric nurses) will review the original manual and adapt it for use by lay health workers in the Ugandan setting. At the second workshop, the locally adapted PST-PC manual will be used to train potential lay health workers (including members of the village health team, representatives of patient support groups and hospital ward attendants), with the feedback obtained during the workshop used to further refine the PST-PC manual. After incorporating the feedback from the second workshop, the PST-PC manual will translated into the local language (Luganda) by a bi-lingual psychologist.

#### 3) To evaluate the acceptability, feasibility, and impact on mental health outcomes of the Ebola+D mental health intervention.

**Fig 1:**
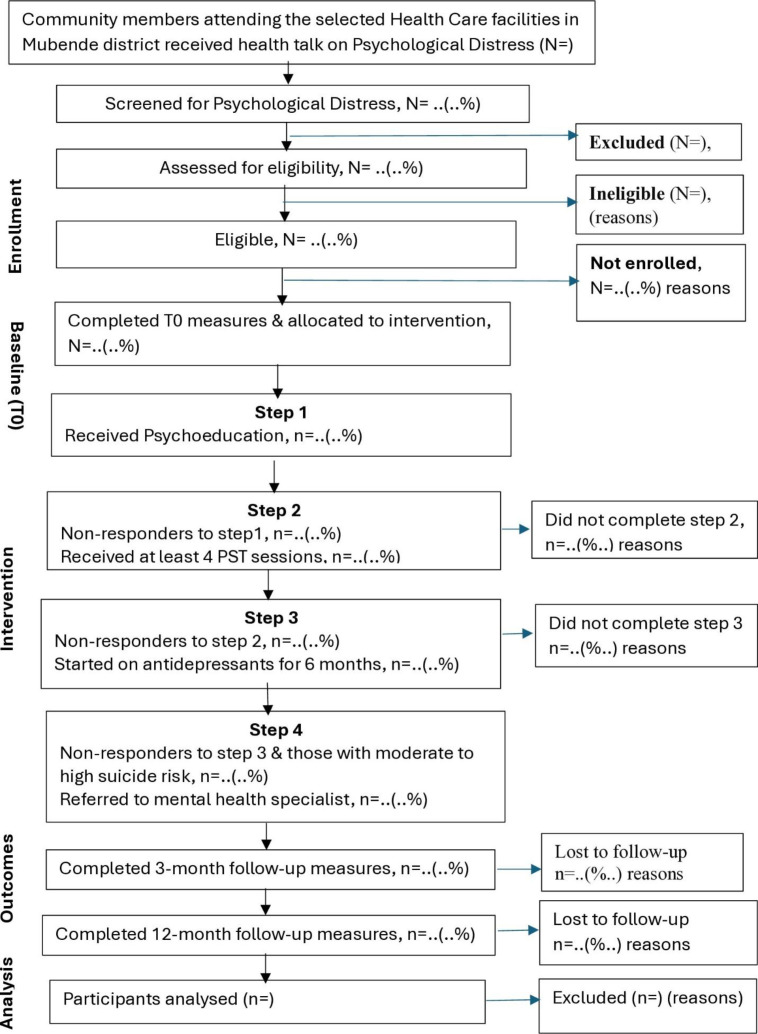
Ebola+D study flowchart.

At the study PHCFs, community members accessing mental health services will be screened using the WHO SRQ-20. Those meeting the eligibility criteria, including ‘significant psychological distress’ (SRQ-20 scores ≥6) [29], will be triaged into appropriate levels of mental health treatment within the Ebola+D intervention. The triage process will consider baseline SRQ-20 scores, a suicidality risk assessment (based on a positive response to SRQ-20 item 16: ‘Do you feel that you are a worthless person?’ or item 17: ‘Has the thought of ending your life been in your mind?’), and confirmation of high suicide risk by the Supervisor (**Fig 1**).

Eligibility criteria for participating in this study: i) Community member of Mubende district staying within the catchment area of the PHCF; ii) 18 years and above, iv) able to communicate in either English or Luganda (local language spoken in the study region and the language into which the questionnaires will be translated), v) has a WHO SRQ-20 score of 6 and above.

Exclusion criteria: i) unable to engage with the research process for any reason that may include sensory impairment or cognitive impairment.

The study aims to enrol at least 100 respondents with psychological distress (SRQ-20 score ≥ 6) from each of the 11 participating PHCFs. Enrolled respondents will be provided with PST at bi-weekly sessions. Monthly assessments will be undertaken to monitor their level of psychological distress using the SRQ-20 until psychological distress scores have fallen to less than 6. A patient will be declared in remission if after completing their prescribed therapy (PST or medication) they remain symptom free (SRQ-20 scores of less than 6) on two occasions one month apart. A full complement of supporting and resource materials including clinic posters, health worker aide-memoire, and patient and care-giver resource materials will be developed and evaluated. The reporting of the trial will be in accordance with the SPIRIT guidelines for interventional trials [40] (S1 File).

### Sample size and power calculations

The study will be longitudinal, with participants followed over a 12-month period. Psychological distress will be assessed using the SRQ-20, with a score of 6 or higher indicating distress. The primary outcome of interest will be remission of distress, defined as an SRQ-20 score below 6. All participants enrolled at baseline will have a level of distress. The intervention is hypothesised to reduce distress prevalence by at least 25%.

Given the longitudinal design and the use of clinic-based interventions, clustering effects will be accounted for by applying a design effect of 1.2, which reflects improved clustering control. Statistical parameters will include a confidence level of 95%. The total sample size will be fixed at 1,000 participants, requiring adjustments to the effective sample size to account for clustering.

The power calculation will be performed using a z-test for two proportions to compare the baseline and post-intervention prevalence of distress.

Using the assumed 25% absolute reduction in distress prevalence, the adjusted effective sample size of 1,000 participants, and a two-tailed significance level of 0.05, the estimated power of the study is calculated to be 94.5%.

Given the 12-month follow-up period and the community-based nature of the intervention, we anticipate an attrition rate of approximately 15%, based on evidence from similar longitudinal studies in rural Ugandan settings. Despite the anticipated attrition, the study will maintain the recruitment target at 1,000 participants, acknowledging that some loss to follow-up is expected. The analysis will account for missing data through multiple imputation techniques and sensitivity analyses to ensure that the study maintains sufficient power and validity to detect the hypothesized effect size.

#### 4) To examine the costs of the Ebola + D mental health intervention.

A subset (n = 6) of the Ebola +D study facilities will be selected for the provider costing using a multi-stage stratified random sampling approach based on facility level and catchment population. We will estimate the average total unit cost per patient for screening and treatment of mental health disorders at the health facilities in Mubende district. A combination of retrospective and prospective methods of cost data collection will be used. Both macro (top-down) and micro (bottom-up) costing approaches will be applied. The disaggregated provider costs will be summed to produce a unit cost per patient for screening and treatment of mental health problems from the provider perspective.

#### 5) To explore the Ebola virus disease (EVD) associated negative beliefs and lived out experiences of affected members of the community.

A qualitative sub-study will be undertaken to explore the Ebola virus disease (EVD) associated negative beliefs and lived out experiences of affected members of the community. Fifty-nine (59) participants will be purposively sampled and stratified by gender from different categories of community members: patients/survivors (n = 15); suspected cases who were kept in the Isolation Unit (n = 5); family members of survivors and those who lost lives (n = 15); community members (including opinion leaders and traditional healers) (n = 8), health workers (n = 8); and (iv) volunteers (n = 8). The interviews will elicit participants’ perspectives on their experience living through the EVD epidermic, how it affected them psychosocially, how they coped with the psychosocial challenges and their continued psychosocial needs.

### Data collection, management, and analyses

#### i) To adapt the HIV + D collaborative stepped care mental health intervention to produce the Ebola+D mental health intervention.

Data will be collected from a number of sources, including case notes and transcripts from the audio recordings of the ToC workshops, existing documentation from the Ministry of Health department of Mental Health and Substance Use as well as minutes of the Ebola+D research meetings with Mubende health officials. For the qualitative data collected, content analysis will be undertaken after coding the transcripts; with the coding categories directly derived from the content of transcripts, without imposing any preconceived theoretical perspectives. A Qualitative data analysis software (nVivo_14_) will be used to aid the data analysis process.

#### ii) To adapt and translate the Problem Solving Therapy for Primary Care (PST-PC) treatment manual to the local rural situation in Mubende district.

Presentations at the plenary sessions will be audio recorded and transcribed. Flip chart records from the different groups will also be collected. The resultant qualitative data will be analysed using thematic analysis techniques [41] and used to improve the revised draft PST-PC training materials.

#### iii) To evaluate the acceptability, feasibility, and impact on mental health outcomes of the Ebola+D mental health intervention.

**Fig 2:**
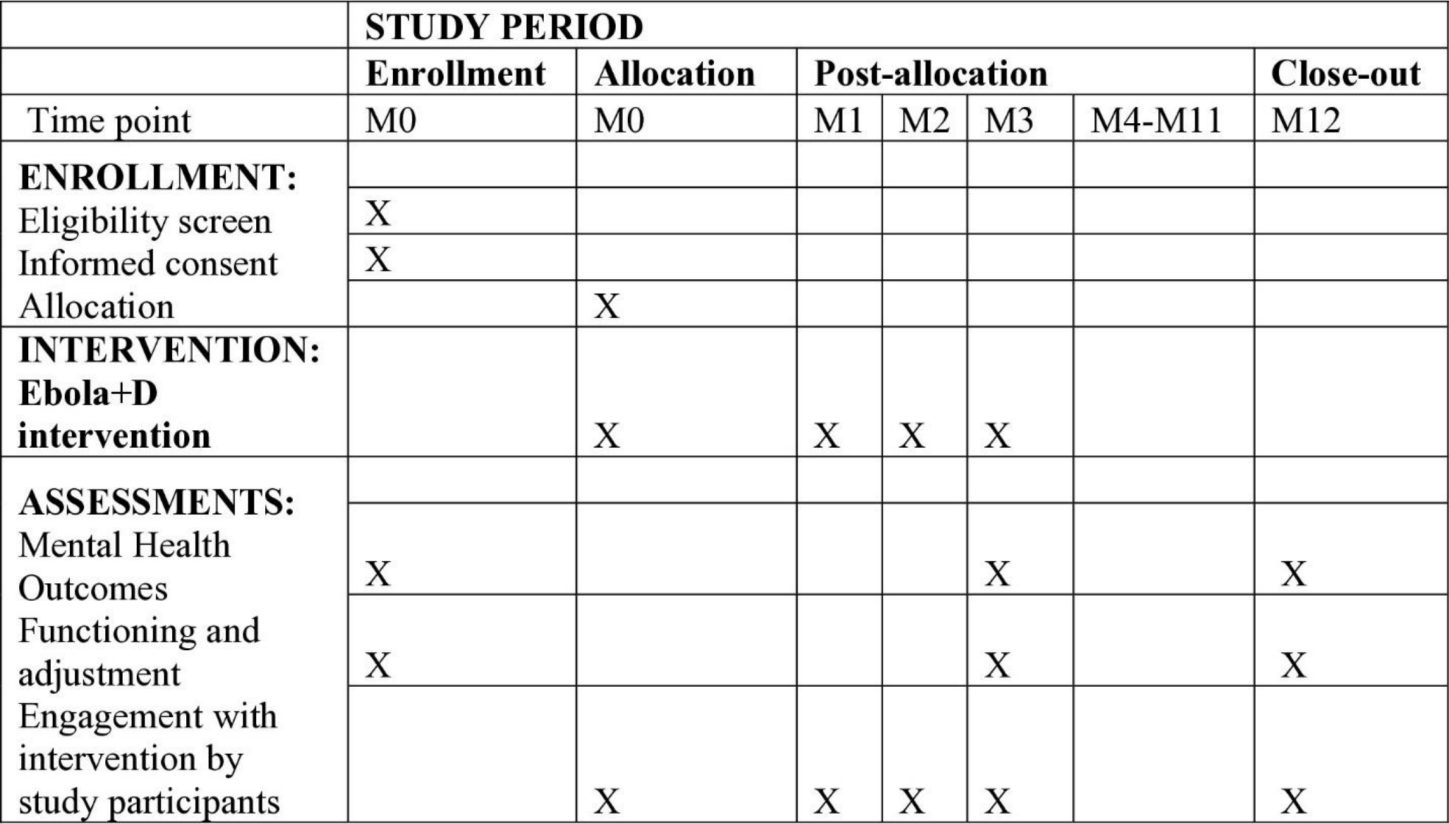
Schedule of study activities. M, month. The questionnaire will assess for: psychological distress, using the SRQ-20 scores [29], depression, using the Patient Health Questionnaire (PHQ-9) [42,43], generalized anxiety symptoms, using the Generalized Anxiety Disorder 7 (GAD-7) [44], alcohol abuse disorder, using the Alcohol Use Disorders Identification Test (AUDIT) [45], suicidality, using the suicidality module in the Diagnostic and Statistical Manual of Mental Disorders (DSM) based structured interview, the M.I.N.I. neuropsychiatric interview (MINI Plus) [46], post-traumatic stress disorder, using the PTSD Checklist for DSM-5 (PCL-5) [47], social support, using the Multidimensional Scale of Perceived Social Support (MSPSS) [48], and degree of disability, using the Sheehan Disability Scale (SDS) [49]. Participants’ engagement with PST will be assessed after each session by the lay health worker using Stahl and colleagues (2017) Interventionists’ Rating Scales of Participants’ Engagement in PST [50]. This instrument has three scales: participation scale (one item), understanding of materials scale (one item), and homework effort scale (one item). Each of these scales is rated on a Likert scale ranging from 1(none) to 6 (excellent). Carers’ (lay health workers, supervisors, mental health specialists) perceptions and satisfaction levels with integration efforts at their clinic will be assessed using a modified Staff Survey adapted from Ede and colleagues, 2015 [51]. Monthly Health Management Information Systems (HMIS) records on mental health diagnoses of each PHCF clinic will be collected. A summary of indicators that will be collected is indicated in Table 1.

Serial focus group discussions with lay health workers (n = 11) and in-depth interviews with supervisors (n = 5) and clinicians (n = 5) will be held and the results will be used iteratively to improve the intervention. Data will be collected on the clinical process, including the engagement of patients, ease of implementing the decision support algorithm, barriers experienced in the delivery of the treatment and modifications made. Exit interviews will be held with a sample of both adherent (n = 8) and non-adherent (have dropped out of the intervention) (n = 8) respondents to describe their experiences and reasons for adherence or non-adherence. To assess effectiveness of the mental health intervention, study participants will be interviewed by trained psychiatric nurse research assistants who are not part in clinical care at baseline, 3-months and 12-months using structured standardized questionnaire (**Fig 2**).

**Table 1:** Summary of indicators that will be collected.

Outcome	Definition	Source of Data	Endpoints
Mental Health Outcomes	Psychological distress, by comparing mean SRQ-20 scores at baseline with scores at 3 and 12 months.	SRQ-20 scores [29]	3 months, 12 months
	Depression, by comparing mean PHQ-9 scores at baseline with scores at 3 and 12 months.	PHQ-9 scores [42,43]	3 months and 12 months
	Generalised anxiety, by comparing mean GAD-7 scores at baseline with scores at 3 and 12 months.	GAD-7 scores [44]	3 months and 12 months
	PTSD, by comparing mean PCL-5 scores at baseline with scores at 3 and 12 months.	PCL-5 scores [47]	3 months and 12 months
	Alcohol abuse disorder, by comparing AUDIT scores at baseline with scores at 3 and 12 months	AUDIT scores [45]	3 months and 12 months
	Suicidality, by comparing suicidality module of the M.I.N.I. neuropsychiatric interview (MINI Plus) scores at baseline with scores at 3 and 12 months	Suicidality module of the M.I.N.I. neuropsychiatric interview (MINI Plus) scores [46]	3 months and 12 months
Functioning and adjustment	Degree of functional impairment in the domains of: work/school, social life and home life or family responsibilities	Assessments using the Sheehan Disability Scale (SDS) [52]	3 months and 12 months
	Social support, by comparing the Multidimensional Scale of Perceived Social Support (MSPSS) scores at baseline with scores at 3 and 12 months	Multidimensional Scale of Perceived Social Support (MSPSS) scores [48]	3 months and 12 months
Fidelity of delivery of the intervention by lay health workers	Degree to which an intervention is implemented as described in the protocol.	Audio recording of lay health worker therapy sessions rated using a fidelity assessment scale	3 months during delivery of the therapy
Engagement with intervention by study participants	Participants’ engagement with PST, undertaken after each session, assessment done by lay health worker on three scales: participation scale (one item), understanding of materials scale (one item), and homework effort scale (one item). Each of these scales is rated on a Likert scale ranging from 1(none) to 6(excellent)	Stahl et al., (2017)’s Interventionists’ Rating Scales of Participants’ Engagement in PST [50]	During the administration of the therapy in first 3 months
Engagement with intervention by study participants	Proportion of persons with psychological distress who receive recommended number of treatment sessions	Study records of each patient	3 months
Carers’ perceptions and satisfaction levels with integration efforts at their PHCF	Lay health workers, supervisors, clinicians, mental health specialists’ perceptions and satisfaction levels with integration efforts at their clinic	Assessed using the modified Staff Survey adapted from Ede and colleagues (2015) [51]	3 months
Intermediate health system outcomes	Outcomes related to the uptake and referral at each step of Ebola+D intervention, e.g., proportion who are referred and who subsequently attend each step.	Screening and study logbooks	3 and 12 months
Uptake of mental health services	Number of patients at PHCF who are accessing mental health services	Monthly HMIS records of each participating public health care facility	12 months

Qualitative data will be analysed using thematic analysis techniques. A bottom-up inductive coding approach will be employed to allow codes, patterns and themes to emerge directly from the data. Two researchers will independently code the data. Inter-rater reliability – the extent to which different coders/analysts arrive at similar or consistent observations – will be assessed through regular data analysis team meetings. These meetings will be used to compare coding outputs of the two coders with a purpose of resolving potential discrepancies and generating consensus and refining the coding framework. Process indicators will be collected and analysed using descriptive statistics. Data collection and analysis will be undertaken iteratively and used to improve the intervention in 3 monthly cycles. Preliminary data on the effectiveness of the intervention will be obtained by comparing mean scores of the mental health outcomes at baseline with those at 3 months and 12 months.

Data will be collected electronically using REDCap, a secure, web-based application designed to support Good Clinical Practice (GCP)-compliant data capture. All data entered on REDCap will be transmitted via encrypted (HTTPS/SSL) connections to prevent interception during data transfer. The REDCap database will be hosted on secure servers at the MRC/UVRI & LSHTM Uganda Research Unit, protected by institutional firewalls and regular security monitoring.

Access to the REDCap database will be strictly restricted to authorized study personnel only, based on a role-based access control system. User accounts will require strong passwords and two-factor authentication will be enabled where possible. An audit trail will automatically log all user activities, including data entry, edits, and downloads, ensuring accountability and traceability.

To maintain confidentiality, all study data will be anonymized by assigning unique study IDs to participants at enrolment. Personally identifiable information (PII), such as names and contact details, will be stored separately from research data in password-protected files with restricted access. The linking log between PII and study IDs will be securely stored and accessible only to the Principal Investigator and the data manager. No PII will be included in exported datasets used for analysis.

The data manager to identify data that are missing, inconsistent, or out-of-range, will run internally developed Stata verification/cleaning do-files. The clinic team will perform clinical quality checks to identify potential errors not captured in the automated verification process.

Data will be checked for missingness and sparsity for all the variables. Demographic and psychosocial characteristics of the participants will be summarised using frequencies and percentages for categorical variables, means and medians will be used to summarise continuous variables depending on the nature of the distribution of different types of disorders. The chi-square test and Kruskal Wallis test will be used to assess associations between each of the different factors and the outcome for categorical and continuous variables, respectively. We will use a cut-off of P < 0.1 (2-tailed test) to identify factors for inclusion in the subsequent analysis. To assess the effect of identified social and psychological factors on Remission at 3 months, Relapse at 12 months and Recovery at 12 months, random effects logistic regression models will be fitted. Other factors to consider based on prior literature will include gender, education level, age, and baseline severity of the condition.

### Development of Predictive models for treatment response

Machine learning approaches will be used develop predictive models for treatment response to the 4 different steps of the Ebola+D mental health intervention using demographic, social, psychological and biological (proteins, mRA, genetic) variables that will be collected as part of this research project. We will use supervised machine learning (ML) classification algorithms to fit predictive models among the at-risk group of individuals for the outcome. These models will include Naïve Bayes (NB), Random forests (RF), logistic regression (LR), support vector machines (SVM), extreme gradient boosting (XGBOOST), K-nearest neighbours (KNN) and decision trees (DT). For each model, the model’s performance will then be evaluated using various performance metrics including area under the receiver operating characteristic (ROC) 11curve (AU-ROC), positive predictive value, negative predictive value, specificity, accuracy, precision, recall, and the F1-score. We will use 70% of the available data to train the models and the remaining 30% will be used in the test set. These analyses will be completed using STATA 18 and Python 13.9.2 software versions.

#### iv) To examine the costs of the Ebola+D mental health intervention.

The data collection team will consist of a health economist and a health economics research assistant. Cost data collection will be undertaken over a 3-month period using a standardized Excel based cost data collection tool that will be developed by the research team. Published literature will be reviewed to identify the cost categories used by similar previous studies when developing the tool. In addition to this, the health economics research team will review the Ebola +D mental health intervention documents to develop a cost category list for the program. The developed tool will be piloted in one health facility and tool modifications will be made before costing the remaining 5 health facilities.

### Resources/ Inputs

To determine the costs from the providers’ perspective, the value and quantity of resources/ inputs required to produce outputs like outpatient visits for screening, treatment and follow-up are required. These inputs will be categorized into three, start-up costs, personnel costs and operational costs as provided in Table 2.

**Table 2:** Summary of cost data to be obtained.

Cost Category	Information to be obtained	Data Source
**A. START-UP COSTS**		
**Health Worker Training** Training costs per participant (Lay health workers, clinician and mental health specialist)	- Duration of training (number of days) - Number of health workers trained and their cadre - Transport costs to attend trainings - Accommodation costs - Training materials such as therapy manuals - Costs of hiring training venue - Costs of supplies for training such as pens, notebooks, food - Number of refresher trainings - Costs of trainers (if applicable) - Hiring costs (if applicable)	Project Training Records
**Furniture and Equipment**	Costs of tables, chairs, metallic cabins and umbrellas that will be delivered to the various health facilities.	-Project Records -MRC procurement records
**B. PERSONNEL COSTS**		
**Service delivery costs** Cost for each service delivered by the different health workers involved in the study such as trained lay health workers, clinicians and specialized mental health specialists, phlebotomists	- How many health talks were given? - How many people attended the health talks? - How many patients were screened? - How many patients were enrolled in the study? - How many patients are receiving psychoeducation? - How many patients are receiving problem-solving therapy? - How many times do they review patients receiving treatment? - What is the average time spent delivering treatment to patients? (number of minutes/hours) - How many mental health distress related inpatient bed days were recorded at the facility? - How many patients were suicidal? - How many patients were receiving SSRIs?	-Time study -Facility Data Managers -Intervention Data
**C. OPERATIONAL COSTS**		
**Capital- Building Space** (Costs for space utilized by the Ebola+D intervention)	- Where are health talks given? - Where are the patients screened, enrolled and treated from? - Where are the tests conducted? - How many square meters are the facility? - What is the value of the facility? - What are the maintenance costs?	Direct measurements of building space utilized by the intervention
**Capital – Equipment** (Costs of Equipment utilized by the Ebola+D intervention)	- What equipment are used for the tests carried out? - What is the price of the equipment used? - What are the maintenance costs for the equipment?	-Facility Accountant -Facility Administration records
**Drugs** (Costs of recurrent drugs used in the intervention)	- What drugs are used to manage mental health problems? - What is the origin (producer) - What is the dose and strength of the drug? - What is the mode of administration? - What is the cost of the drug?	National Medical Stores price list
**Supplies** (Costs of recurrent supplies used in the intervention)	- What supplies are used for tests (such as pregnancy tests)? - What supplies are used for screening, enrolment, treatment? - What are the costs of these supplies?	-MRC Project Records -Health Facility Records -National Medical Stores price lists.
**Utilities** (Cost of overhead costs)	What are the recurrent expenditures for electricity, water, internet, telephone/ airtime for the most recent financial year?	Facility Accounts records

Start-up costs include the costs of activities and equipment needed when setting up the intervention (these are a one-off expenditure). Personnel category includes the cost of all staff involved in the delivery of the Ebola+D mental health intervention, while the operational cost category includes the cost of all items that are related to operational activity of the intervention**.**

### Direct costs

Direct medical costs of treatment will be disaggregated and descriptively analysed from baseline to endline. Categories of cost-related data will be assessed to determine the major cost drivers for delivering mental health services for EVD affected communities.

All research costs and start-up costs will be reported separately from the cost of delivering the intervention.

### Provider cost measurement and valuation

The quantity of resources used, and their respective unit costs will be used to calculate the unit cost for each service output such as, outpatient visits (including screening, consultation, treatment and follow-up visits), inpatient bed days and medication. The function below explains the calculation of the service output unit cost.


OUC=n(R) ×p(R)\]

Where OUC is the unit cost per service output, n(R) is the quantity of resources used and p(R) is the unit cost of resources used [53,54].

The total unit cost per patient will be calculated by multiplying the quantity of each service output by the unit cost of the service output from screening to the end of treatment for each patient in the study. The function below explains the calculation of the total unit cost per patient.


TUC=q(S)×OUC\]

Where TUC is the total unit cost per patient, q(S) is the quantity of each service output and OUC is the unit cost per service output [53,54].

The average cost per patient for screening and treatment will be derived and compared across 6 facilities using a t test.

### Time study

In addition to the data that will be collected on personnel salary and benefits from the facility records, we will conduct a time study to capture the proportion of each staffs’ time allocated to the intervention [55,56]. We shall develop an activity log with a list of duties such as delivering health talks, delivering psychoeducation, problem solving therapy and drug prescription that will be prospectively completed by intervention staff to record their time spent on Ebola+D related activities.

### Sensitivity analysis and uncertainty

We shall carry out a deterministic sensitivity analysis to deal with uncertainty and examine the robustness of the Ebola+D intervention costs at the extreme points [53]. We shall use the scenario analysis where we will calculate program costs using two scenarios, the most expensive and least expensive case to see how cost of the program changes. We shall first identify the major cost components of the intervention that are likely to change overtime and vary across different settings. For example, for the Ebola+D mental health intervention, personnel costs such as salaries of the lay health workers will be varied with highest and lowest salary level.

#### v) To explore the Ebola virus disease (EVD) associated negative beliefs and lived out experiences of affected members of the community.

Data collection will be conducted at the health facility/EVD Treatment Unit or at a location preferred by the participant. With the permission of the participant, the interviews will be voice-recorded and subsequently transcribed verbatim. An interpretative phenomenological analysis of data will be done.

### Risk assessment and management

There are no known side effects of psychological treatments including PST that will be used in this project. There are however important adverse effects that could arise from taking selective serotonin re-uptake inhibitors (SSRIs) such as fluoxetine that include side effects of the antidepressants themselves [57] and drug to drug interactions between the antidepressants and other treatments for chronic illnesses such as antiretroviral therapy for HIV/AIDS [58]. The clinicians and mental health specialists participating in this study, who will be responsible for prescribing SSRIs, will undergo orientation training to update them on potential adverse events.

Due to safety concerns (although the evidence is still ambiguous) about the use of fluoxetine (an SSRI) in pregnant women [59], we shall be guided by the following: i) All women enrolled into this study who require an SSRI will have to undertake a pregnancy test, and if found to be positive shall only be offered the option of psychotherapy (PST); ii) Pregnant non-responders to PST will be referred directly to specialist mental health workers (who will treat them according to national guidelines); iii) the study will follow the same procedure for nursing mothers (lactating mothers); iv) Women on antidepressant medication who become pregnant during the course of the study will be offered a choice to withdraw from the antidepressant with an explanation of the risks and benefits and, if needed, offered PST.

### Study monitoring and auditing

An advisory committee will monitor the progress of the study and details are shown in appendix 1 (S1 Appendix 1-Study advisory committee).

Auditing by the Sponsor and inspection by the ethics committees will be undertaken.

### Ethical considerations and dissemination

The protocol was reviewed and approved by the Uganda Virus Research Institute (UVRI) Research and Ethics Committee (reference number GC/127/961), the Uganda National Council for Science and Technology (reference number HS2870E), and the London School of Hygiene and Tropical Medicine (LSHTM) Ethics Committee (reference number 29605). The study sponsor is London School of Hygiene and Tropical Medicine through Medical Research Council (MRC)/Uganda Virus Research Institute (UVRI) and London School of Hygiene and Tropical Medicine (LSHTM) Uganda Research Unit. Written (or witnessed if the participant is illiterate) informed consent will be obtained from each potential participant before enrolment into the study. All participant data will be stored securely, and access will be restricted. To maintain participant confidentiality, all study data collection forms will only be identified by the study identification numbers. Documents that contain participant’s names such informed consent forms will be stored separately from data collection forms. All databases will be password protected. Any protocol modifications will be submitted to the UVRI Research and Ethics Committee for review, and participants will be informed if warranted. Respondents found to have significant psychiatric problems will be referred to the nearest health facility for treatment. Serious Adverse Events (SAEs) from any cause will be reported as soon as possible within seven calendar days of becoming aware of SAE to the UVRI Research and Ethics Committee and to the advisory committee. Results produced by this investigation will be presented at local and international conferences and published in a timely fashion, ideally in the last year of the study period. All final peer-reviewed manuscripts that arise from this proposal will be submitted to the digital archive PubMed Central for open access.

## Discussion

The aim of the Ebola+D project is to develop an intervention model to address the medium- to long-term Ebola associated psychological Distress and psychosocial problems in public health facilities in low resourced settings such as those in Mubende District in central Uganda. This intervention aims to provide a model that could be used to address the medium- and long- term mental health consequences of outbreaks of Emerging Viral Diseases in remote and poorly resourced settings in LMIC. The proposed model is a collaborative stepped care approach modelled on the HIV + D mental health intervention (a depression integration model in routine HIV care) [17] which was developed by the Mental Health Section based on the MANAS intervention that demonstrated effectiveness and cost-effective in primary care in India [18]. This collaborative care model will be led by lay health care workers who will administer a manualised Problem Solving Therapy under the supervision of nurses or counsellors based at primary level public health care facilities and supported by mental health workers based at the regional referral hospital.

## Supporting information

S1 File SPIRIT 2013 checklist. (PDF)

S1 Appendix Study advisory committee. (PDF)

S2 File Approved study protocol. (PDF)

## Abbreviations

bola+D: Acronym for the project ‘Proposal to address the medium- to long-term EBOLA associated psychological Distress and psychosocial problems in Mubende District in central Uganda’
EVD: Ebola Virus Disease
PST: Problem Solving Therapy
PST-PC: Problem Solving Therapy for Primary Care
LHW: Lay health worker
HIV + D: Acronym for the project, ‘Integrating the management of depression into routine HIV care in Uganda
LMIC: Low and middle income countries
MANAS: Acronym for the cluster randomised trial that was implemented in India to evaluate a stepped care collaborative delivery model for depression and anxiety disorders in primary health care
WHO: World Health Organisation
MOH: Ministry of Health
MRC/UVRI & LSHTM Uganda Research Unit: Medical Research Council/ Uganda Virus Research Unit & London School of Hygiene and Tropical Medicine Uganda Research Unit
PHCF: Public health care facilities
ToC: Theory of Change
UNCST: Uganda National Council for Science and Technology
UVRI: Uganda Virus Research Institute
REC: Research Ethics Committee
