# Supplementary material for: Protocol to develop and pilot a primary mental healthcare intervention model to address the medium- to long-term Ebola associated psychological distress and psychosocial problems in Mubende District in Central Uganda (the Ebola+D project)
Source: PLoS One. 2025 Aug 6;20(8):e0329591. doi: 10.1371/journal.pone.0329591 (PMC12327640; doi:10.1371/journal.pone.0329591)
Supplement: S1 Appendix — (PDF) [file pone.0329591.s002.pdf]

Appendix 1. Study advisory committee

Role	Members	Frequency of meeting
To receive and review information on the progress and accruing data (including serious adverse events) and provide advice on the conduct of the study.	1) Prof Crick Lund, Professor of Global Mental Health, Health Service and Population Research Department, Institute of Psychiatry, Psychology and Neuroscience, Kings College London and Professor of Public Mental Health, Department of Psychiatry and Mental Health, University of Cape Town.<br><br>2) Prof. Wilson Muhwezi, Social Anthropologist, Department of Psychiatry, Makerere University.<br><br>3) Dr. Hafsa Sentongo, Ag. Assistant Commissioner Mental Health and Control of Substance Use, Ministry of Health.<br><br>4) Dr Kenneth Kalani, Psychiatrist, Mental Health and Control of Substance Use, Ministry of Health.<br><br>5) Dr. Giulia Greco, Centre for Health Economics, London School of Hygiene & Tropical Medicine<br><br>6) Prof. Valeria Mondelli, Maurice Wohl Clinical Neuroscience Institute, Kings College.<br><br>7) Dr Emmanuel Batibwe, Director Mubende Regional Referral Hospital<br><br>8) Prof Birthe N Knizek, Department of Mental Health, Norwegian University of Science and Technology, Norway.<br><br>9) Dr Nambusi Kyegombe, Head of Social Sciences, MRC/UVRI & LSHTM Uganda Research Unit	Six monthly intervals
