# Supplementary material for: Protocol to develop and pilot a primary mental healthcare intervention model to address the medium- to long-term Ebola associated psychological distress and psychosocial problems in Mubende District in Central Uganda (the Ebola+D project)
Source: PLoS One. 2025 Aug 6;20(8):e0329591. doi: 10.1371/journal.pone.0329591 (PMC12327640; doi:10.1371/journal.pone.0329591)
Supplement: S2 File — (PDF) [file pone.0329591.s003.pdf]

Proposal to address the medium- to long-term EBOLA associated psychological Distress and psychosocial problems in Mubende District in central Uganda (Ebola+D Project)

Principal Investigator:

Prof. Eugene Kinyanda,

Head, Mental Health Section,

MRC/UVRI & LSHTM Uganda Research Unit

Confidentiality Statement

This document contains confidential information that must not be disclosed to anyone other than the Sponsor, the Investigator Team, the Host Organisation, and members of the Research Ethics Committee, unless authorised to do so. This information cannot be used for any purpose other than the evaluation or conduct of the study without the prior written consent of Prof Eugene Kinyanda.

Co-Investigators:

Dr. Richard S. Mpango, Clinical Psychologist, Mental Health Section, MRC/UVRI & LSHTM Uganda Research Unit

Dr. Leticia Kyohangirwe, Psychiatrist, Mental Health Section, MRC/UVRI & LSHTM Uganda Research Unit

Dr. Rwamahe Rutakumwa, Senior Social Scientist, Mental Health Section, MRC/UVRI & LSHTM Uganda Research Unit

Mr. Joshua Ssebunnya, Clinical Psychologist, Uganda Coordinator of the PRIME consortium

Ms. Barbra Kiconco, Health Economist, Mental Health Section, MRC/UVRI & LSHTM Uganda Research Unit

Ms. Christine Tusiime, Clinical Psychologist, Butabika National Psychiatric Referral Hospital

Mr. Isaac Sekitoleko, Statistician, Mental Health Section, MRC/UVRI & LSHTM Uganda Research Unit

Mr. Wilber Ssembajjwe, Data Manager, Mental Health Section, MRC/UVRI & LSHTM Uganda Research Unit

Dr. Andrew Obuku, Immunologist, Basic Science Programme, MRC/UVRI & LSHTM Uganda Research Unit

Collaborators:

Prof. Wilson Muhwezi, Social Anthropologist, Department of Psychiatry, Makerere University
Dr. Hafsa Sentongo, Ag. Assistant Commissioner Mental Health and Control of Substance Use, Ministry of Health
Dr Kenneth Kalani, Psychiatrist, Mental Health and Control of Substance Use, Ministry of Health
Prof Vikram Patel, Department of Global Health and Social Medicine, Harvard Medical School
Prof Ricardo Araya, Centre for Global Mental Health, Kings College
Dr. Giulia Greco, Global Health Economics Centre. LSHTM
Prof Crick Lund, Centre for Global Mental Health, Kings College
Prof. Valeria Mondelli, Maurice Wohl Clinical Neuroscience Institute, Kings College
Dr Emmanuel Batibwe, Director Mubende Regional Referral Hospital
Prof Birthe N Knizek, Department of Mental Health, Norwegian University of Science and Technology
Dr Nambusi Kyegombe, Head of Social Sciences, MRC/UVRI & LSHTM Uganda Research Unit

General Sponsor Information

**Sponsor Protocol Number**	RGEK230305
**Registration of Study**	TBC
**Product of study/Intervention**	Psychosocial Intervention
**Sponsor**	London School of Hygiene and Tropical Medicine Through Medical Research Council (MRC)/Uganda Virus Research Institute (UVRI) and London School of Hygiene and Tropical Medicine (LSHTM) Uganda Research Unit
**Funding**	Medical Research Council (UK)
**Principal Investigator**<br>Address:<br>Email:<br>Phone:	Prof Eugene Kinyanda<br>MRC/UVRI & LSHTM Uganda Research Unit<br>Eugene.Kinyanda@mrcuganda.org<br>+256 788461950
**Local Sponsor Contact**<br><br><br><br><br><br><br><br>**Sponsor Contact**	Research Governance Department (RGD)<br>MRC/UVRI & LSHTM Uganda Research Unit<br>Plot 51-59, Nakiwogo Road<br>P.O.Box 49, Entebbe (Uganda)<br>Tel: +256417704191<br>Email: regulatory@mrcuganda.org<br><br><br>Research Governance and Integrity Office (RGIO)<br>London School of Hygiene & Tropical Medicine<br>Keppel Street<br>London WC1E 7HT<br>Tel: +44 207 927 2626<br>Email: RGIO@lshtm.ac.uk
**Indemnity:**<br><br><br><br>**Monitoring**<br><br>**Auditing**	The Sponsor guarantees availability of Public Liability ("negligent harm") and Clinical Trial ("non-negligent harm") insurance policies which apply to this trial<br><br>To be implemented and coordinated by the Local Sponsor<br><br>To be implemented and coordinated by the Sponsor

Signature Page

By my signature below, I hereby confirm that I will conduct the study described in this protocol in compliance with ICH/GCP and the version of such protocol agreed to by the applicable regulatory authorities and approved by all Institutional Review Boards/Ethical Committees.

Prof Eugene Kinyanda (Principal Investigator)

______________________________ ________________

Signature Date

Dr. Kimbugwe Geofrey (Local Sponsor Representative)

______________________________ ________________

Signature Date

Table of Contents

General Sponsor Information 3

Signature Page 4

1.0 SUMMARY 6

2.0 ABBREVIATIONS 7

3. INTRODUCTION 8

3.1 Background 8

3.2 Mental Health and Psychosocial Support Services in Mubende District 10

3.3 Proposal to address the medium- to long-term EBOLA associated psychological Distress and psychosocial problems in Mubende District in central Uganda (Ebola+D) 11

4.0 RESEARCH COMPONENT 14

4.1 SUB-COMPONENT I: TO DEVELOP, IMPLEMENT AND EVALUATE THE EBOLA+D MENTAL HEALTH INTERVENTION IN MUBENDE DISTRICT 15

4.1.1. General Objective: 15

4.2 SUB-COMPONENT II: TO INVESTIGATE THE NATURE OF MENTAL HEALTH PROBLEMS OF COMMUNITY MEMBERS ACCESSING THE EBOLA+D MENTAL HEALTH INTERVENTION AND FACTORS ASSOCIATED WITH TREATMENT OUTCOMES (EBOLA+D COHORT SUB-COMPONENT) 29

4.2.1 General Objectives: 29

4.3 SUB-COMPONENT III: TO EXPLORE THE EVD ASSOCIATED NEGATIVE BELIEFS AND LIVED OUT EXPERIENCES OF AFFECTED MEMBERS OF THE COMMUNITY (EBOLA+D QUALITATITIVE SUB-COMPONENT) 42

5.0 ETHICAL CONSIDERATIONS, MONITORING AND AUDITING 43

6.0 PANEL OF EXPERTS 44

7.0 REFERENCES 44

8.0 APPENDICES 50

1.0 SUMMARY

Background: The Ebola Virus Disease (EVD) can lead to life-threatening multiple system disease. To-date Uganda has had seven Ebola outbreaks and the just ended 2022 Ebola outbreak in Uganda involved nine districts (Mubende Kassanda, Kyegegwa, Masaka, Wakiso, Bunyangabu, Jinja, Kagadi, Kampala), with 142 confirmed cases, 55 confirmed deaths, 87 recoveries, an additional 22 probable deaths (in individuals who died before samples could be taken), 19 healthcare workers (of whom seven died) and over 4,000 contacts followed up. EVD in addition to being associated with high mortality and physical morbidity among patients and survivors, is associated with significant mental health and psychosocial problems among patients/survivors, their family members, members of the affected communities, health workers and volunteers. In response to the EVD epidemic in the district, Mubende saw an influx of different cadres of responders including members of the Mental Health and Psychosocial Support Services who supported the district to address the immediate mental health and psychosocial effects of EVD. Recent literature suggests that the mental health consequences of EVD persist much longer after the epidemic. To support the medical services of Mubende district cope with the Ebola associated mental health and psychosocial problems, the Ministry of Health of Uganda has partnered with the MRC/UVRI & LSHTM Uganda Research Unit to implement a health systems strengthening project in the district entitled, *'Proposal to address the medium- to long-term EBOLA associated psychological Distress and psychosocial problems in Mubende District in central Uganda (Ebola+D Project)'.*

Objectives: To undertake a project to address the medium- to long-term EBOLA associated psychological Distress and psychosocial problems in Mubende District in central Uganda**.**

Methodology: The project will be undertaken in the 11 selected health care facilities in Mubende district. Members of the community attending these selected health care facilities will be screened for psychological distress using the locally validated Luganda or English version of the WHO- Self Report Questionnaire (SRQ-20). Those individuals found to have significant psychological distress (a score of 6 and above) will be offered Ebola+D intervention. The intervention will include psycho-education, Problem Solving Therapy, antidepressant medication and referral to a supervising specialist mental health worker. A cohort of EVD affected

individuals accessing the Ebola +D mental health intervention will be recruited and followed up for 12 months with assessments at three time points; baseline, 3 months and 12 months.

Results: This project will address the medium- to long-term Ebola associated psychological Distress and psychosocial problems and it will also determine the nature of the mental health problems among the community members affected by the Ebola epidemic.

Utility: The study findings will inform future mental health and psychosocial interventions in Ebola affected communities in Africa.

2.0 ABBREVIATIONS

EBOLA+D: Acronym for the project, 'Proposal to address the medium- to long-term EBOLA associated psychological Distress and psychosocial problems in Mubende District in central Uganda'

EVD: Ebola Virus Disease

MRRH: Mubende Regional Referral Hospital

MHPSS: Mental Health and Psychosocial Support Services

VHT: Village Health Team

HIV+D: Acronym for the project, 'Integrating the management of depression into routine HIV care in Uganda'

MANAS: Acronym for the cluster randomised trial that was implemented in India to evaluate a stepped care collaborative delivery model for depression and anxiety disorders in primary health care.

PST: Problem Solving Therapy

PST-PC: Problem Solving Therapy for Primary Care

PTSD: Post-traumatic stress disorder

SRQ-20: Self Report Questionnaire

LMIC: Low and middle income countries

mhGAP: WHO's Mental Health Gap Action Programme

ToC: Theory of Change

UVRI: Uganda Virus Research Institute

MOH: Ministry of Health

PHCFs: Public health care facilities

PHQ-9: The Patient Health Questionnaire-9

GAD-7: Generalised Anxiety Disorder-7

AUDIT: Alcohol Use Disorders Identification Test

DSM: Diagnostic and Statistical Manual of Mental Disorders

PCL-5: PTSD Checklist for DSM-5

VHT: Village Health Team

UNCST: Uganda National Council for Science and Technology

3. INTRODUCTION

3.1 Background

The Ebola Virus Disease (EVD), belonging to the filoviruses family is one of the causes of viral haemorrhagic fever that can lead to life-threatening multiple system disease[1]. EVD first appeared in 1976 in two simultaneous outbreaks, one in Nzara (South Sudan), the other in Yambuku (Democratic Republic of the Congo)[1]. The 2014–2016 Ebola outbreak in West Africa was the largest outbreak since the virus was first discovered, affecting 10 countries with a total of 28, 600 people infected and 11, 325 dead[2]. To-date Uganda has had seven Ebola outbreaks, five outbreaks due to the Sudan Virus Disease (SUVD) 2000, 2011, 2012 (two outbreaks) and 2022 (the current outbreak); one outbreak due to the Bundibugyo virus disease (BVD) in 2007; and one outbreak due to Ebola Zaire disease (EZD) in 2019[3]. The just ended 2022 Ebola outbreak in Uganda involved nine districts (Mubende Kassanda, Kyegegwa, Masaka, Wakiso, Bunyangabu, Jinja, Kagadi, Kampala), with 142 confirmed cases, 55 confirmed deaths, 87 recoveries, an additional 22 probable deaths (in individuals who died before samples could be taken), 19 healthcare workers were infected (of whom seven died) and over 4,000 contacts followed up[4].

EVD in addition to being associated with high mortality and physical morbidity among patients and survivors, is associated with significant mental health and psychosocial problems among patients/survivors, their family members, members of the affected communities, health workers and volunteers[5-11]. Psychosocial problems that have been reported to be associated with EVD

include loss of loved ones, rejection, isolation, stigmatisation, threatened violence and sometimes actual violence, loss of employment and trauma associated with EVD illness experience (including the manner of evacuation from home that involves destruction of personal effects to prevent transmission of disease, and the harrowing experience in the Ebola Treatment Unit)[5,7,8]. The mental health problems associated with Ebola include the formal mental health disorders of depression, anxiety disorders and post-traumatic stress disorder (PTSD)[5,6,7,8]. Additional mental health problems include anger, grief, guilt, flashbacks, suicidal tendencies, alcohol addiction, self-stigmatisation and sleep difficulties[5,8]. Since the West African EVD epidemic, there is growing evidence for possible central nervous system (CNS) viral invasion including clinical and imaging features suggestive of meningoencephalitis and meningitis, explaining the residual neurocognitive impairment that has been reported to be associated with EVD[8,9,12,13].

For the health workers, providing medical care for EVD- infected patients is psychologically very stressful due to treating very sick patients, high mortality in the Ebola Treatment Units, extended shift times, risk of infection, fear of infecting family members and witnessing the death of colleagues[10]. In addition, the wearing of personal protective equipment is uncomfortable, impairs communication and performance of diagnostic and therapeutic procedures[10]. All these predispose these frontline health workers and volunteers to mental health and psychosocial problems including anxiety, depression, fatigue and social isolation[10]. If these mental health problems and associated psychosocial problems are not addressed, these will lead to impairment in quality of life, loss of productivity, secondary psychosocial problems such alcohol abuse and suicidality and tendency to chronicity as reported in one study where mental health problems were reported to have persisted for over two decades among survivors of EVD[9,11]. At the community level, inattention to the psychosocial challenges and misperception may lead to inter-community violence.

To address, these mental health and psychosocial challenges, Ebola National Task Force created an Ebola Mental Health and Psychosocial Support Services (MHPSS) sub-pillar headed by Dr Hafsa Lukwata Sentongo, Ag. Assistant Commissioner Mental Health and Control of Substance Use, with Prof Eugene Kinyanda, head of the Mental Health Section at the MRC/UVRI & LSHTM Uganda Research Unit a member. The MHPSS sub-pillar articulated three broad objectives which guided the response in this area:

Objective 1: Build capacity of health workers and community volunteers to provide mental health and psychosocial support

Objective 2: Provide mental health and psychosocial support services to the community (EVD survivors, suspected cases, affected families, grieving families, affected communities)

Objective 3: Provide mental health and psychosocial support services to frontline health workers

3.2 Mental Health and Psychosocial Support Services in Mubende District

Mubende district (population 720,000) even at the best of times before the Ebola epidemic has had a very fragile mental health service system that consisted only of a mental health department at Mubende Regional Referral Hospital (MRRH). The entire district mental health work force consists of only 12 mental health workers (1 psychiatrist, 2 psychiatric clinical officers and 9 psychiatric nurses) all based at the regional referral hospital (*Dr Kenneth Kalani, field coordinator of the Ebola MHPSS response- personal communication December 2022*). Apart from MRRH, the district has other public health care facilities at the levels of Health Centre IIs and IIIs that are spread out in the district managed by general clinicians and nurses. The first level of primary health care service provision in the Uganda health system is the Village Health Team (VHT) or Health Centre I who are supervised by a Health Assistant based at a Health Centre II or III.

In response to the EVD epidemic in the district, Mubende saw an influx of different cadres of responders including members of the Mental Health and Psychosocial Support Services who supported the district to address the immediate mental health and psychosocial effects of EVD. Recent literature suggests that the mental health consequences of EVD persist much longer after the epidemic has cleared including in one study that reported that these had persisted for over two decades[9]. In preparation for the scale down of MHPSS, the Ministry of Health with support of partners trained 90 members of the Village Health Team (10 per sub-county) to provide continued mental health and psychosocial support to the district. But for these trained VHTs to provide effective psychosocial support will require continued supervision and upward linkage to

more specialised mental health services at the regional referral hospital, a component that is lacking.

3.3 Proposal to address the medium- to long-term EBOLA associated psychological Distress and psychosocial problems in Mubende District in central Uganda (Ebola+D)

To support the medical services of Mubende district cope with the Ebola associated mental health and psychosocial problems, the Ministry of Health of Uganda has partnered with the MRC/UVRI & LSHTM Uganda Research Unit to implement a health systems strengthening project in the district entitled, *'Proposal to address the medium- to long-term EBOLA associated psychological Distress and psychosocial problems in Mubende District in central Uganda (Ebola+D Project)'.*

Ebola +D Mental Health intervention

The proposed Ebola +D mental health intervention will aim to address the three most commonly reported medium- to long- term mental consequence of EVD, namely: depression, anxiety disorders and post-traumatic stress disorder in EVD affected members of the community (EVD survivors, suspected cases- contacts of Ebola cases who were put in isolation until they were declared EVD free and then returned to the community, grieving family members, other affected community members). Ebola+D will employ a collaborative stepped care approach modelled on the HIV+D mental health intervention[14] which was developed by the Mental Health Section based on the MANAS intervention that demonstrated effectiveness and cost-effective in primary care in India[15]. The HIV+D mental health intervention is a collaborative stepped care mental health intervention model that was developed and is currently under trial through a Senior Wellcome Trust funded study to provide depression management to adults living with HIV/AIDS attending public health care facilities in Uganda[14]. Preliminary analyses of HIV+D trial has shown effectiveness of the HIV+D mental health intervention against depression and generalised anxiety at 3 and 12 months (proceedings of the 21st-23rd HIV+D Data analysis workshop).

The Ebola +D intervention will be guided by four principles: i) the intervention should address the health system challenges in the district (low mental health literacy in the community and among general health workers; severe shortage of mental health workers in the district; shortage of other cadres of health care workers)[16,17]; ii) use the best global practices hence the task-shifting approach of using supervised trained lay health workers to deliver low intensity psychological treatments[15,18,19] ; iii) the selected clinical treatments should have been shown to be effective against the target mental disorders (depression, anxiety disorders and PTSD), hence the selection of the psychoeducation[20], the transdiagnostic Problem Solving Therapy (PST)[21], and the use of Selective-Serotonin Re-uptake Inhibitors (SSRIs) for depression, anxiety disorder and PTSD[22-24]; iv) and the intervention should be guided by the needs of the specific patient, aligned with the concepts of person-centred care[25].

The overall goal of the intervention is recovery from the mental health disorders (depression, anxiety and PTSD). This will be guided by two rules: allocation of the clinical treatments based on decision rules defined by severity of symptoms and response; and planned reviews of response at regular intervals (monthly until remission). The locally validated Luganda or English version of the WHO- Self Report Questionnaire (SRQ-20)[26,27] (Luganda is the predominant local language spoken in central Uganda) will be used by the trained lay health workers (members of the Village Health Team) to screen members of the community for psychological distress. The SRQ-20 which was developed by the WHO specifically for low- and middle- income (LMIC) settings employs a yes/no answer format (which is amicable to lay health workers who often have low levels of literary) and is designed to detect non-specific psychological distress, including suicidality[26].

The Ebola +D intervention will be delivered by the health centre based medical team, in partnership with members of the village health team (VHT; the first level of the Ugandan health care system). The intervention will be coordinated by a designated mental health contact person at the participating Health Centre II or III (either a general nurses or general clinician, working at the health facility), and will be supported by mental health professionals based at Mubende Regional Referral Hospital.

The Ebola +D intervention will involve 4 steps:

<u>Step 1 (Initiation of treatment):</u> Patients with SRQ-20 scores of ≥6)[27] are advised about their scores and offered Psychoeducation (undertaken by a lay health worker, member of the VHT)

<u>Step 2: (Management of moderate to severe cases):</u> Patients who remain symptomatic at follow up (SRQ-20 score ≥6, after 4 weeks) despite Step 1. These will be offered Problem Solving Therapy (PST; minimum 4 sessions, maximum 8 sessions) (undertaken by a lay health worker, member of the VHT).

<u>Step 3: (Monitoring outcomes</u>): If after 6 sessions of PST, SRQ-20 scores are still 6 and above, complete PST sessions and add Selective Serotonin Re-Uptake Inhibitor (SSRI; such as Fluoxetine 20mg/day for 6 months) (SSRI medication initiated by clinician).

<u>Step 4: (Referral to Mental Health Specialist/ Clinician in charge of facility</u>): If despite Step 3 there is no improvement (SRQ-20 scores 6 and above); or at initiation of treatment or during any phase of treatment, if someone is deemed to have a high suicide risk confirmed by the supervisor (contact general nurse or clinician), following a positive SRQ-20 item 16 ('do you feel that you are a worthless person?') or/and item 17 ('has the thought of ending your life been in your mind?'), then continue all existing treatment and refer to a specialised mental health worker (psychiatrist, psychiatric nurse or psychiatric clinical officer at Mubende Regional Referral Hospital).

The Ebola +D mental health intervention will be delivered at each of the 11 public health care facilities (Health Centre IIs and IIIs) in the district of Mubende. At each of the 11 public health care facilities, this mental health intervention will be delivered by a team that will include: 2 trained lay health workers (selected from members of the Village Health Team); a Supervisor (selected from the health centre), clinicians (from the health centre); and a visiting specialist mental health worker (psychiatric clinical officer or psychiatric nurse from Mubende Regional Referral Hospital).

The Mental Health Section Research team of MRC/UVRI & LSHTM from their previous experience developing and supporting the HIV+D trial will support the delivery of the Ebola+D mental health intervention in Mubende district. The Mental Health Section will undertake the following: i) develop a Health Talk about the psychosocial and mental health problems associated with EVD to be delivered at the triage area where community members who have

come to access health services at the public health care facilities are waiting (messages will be drawn from the WHO Psychological First Aid Manual[28]); ii) will develop the message that will be given in the Psychoeducation Session (1st step of care in the Ebola+D mental health intervention); iii) will undertake the local adaptation and translation of Problem Solving Therapy for Primary Care (PST-PC) treatment manual[29]; iv) will undertake the training and supervision of lay health workers and their supervisors; v) will undertake the training of clinicians in mhGAP guidelines (including use of SSRIs)[30].

4.0 RESEARCH COMPONENT

A research component will be embedded in this project to inform future mental health and psychosocial interventions in Ebola affected communities in Africa. This research component will have three broad aims: 1) to develop, implement and evaluate the Ebola+D mental health intervention in Mubende district; 2) To investigate the nature of mental health problems of community members accessing the Ebola+D mental health intervention and factors associated with treatment outcomes including immunological biomarkers; 3) To explore the EVD associated negative beliefs and lived out experiences of affected members of the community (including health care workers). To address these three aims, this research project will be divided into three sub-components:

Sub-component I: To develop, implement and evaluate the Ebola +D mental health intervention in Mubende district (Ebola+D Health System sub-component)

Sub-component II: To investigate the nature of mental health problems of community members accessing the Ebola+D mental health intervention and factors associated with treatment outcomes (Ebola+D cohort sub-component)

Sub-component III: To explore the EVD associated negative beliefs and lived out experiences of affected members of the community (Ebola+D qualitative sub-component)

Under each of these sub-components are a number of sub-studies that are described below.

4.1 SUB-COMPONENT I: TO DEVELOP, IMPLEMENT AND EVALUATE THE EBOLA+D MENTAL HEALTH INTERVENTION IN MUBENDE DISTRICT

4.1.1. General Objective:

To adapt the HIV+D c*ollaborative stepped care mental health intervention'*, incorporating evidence based treatments for depression, anxiety and post-traumatic stress disorder (psychoeducation, problem solving therapy and use of selective serotonin re-uptake inhibitors), to be delivered by health facility based health care workers and supervised lay health workers supported by trained mental health workers, to produce the **Ebola+D mental health intervention** (an intervention that address ***Ebola*** related psychological ***D***istress in primary health care). This sub-component will be undertaken in line with the recommendations from the MRC's framework for complex interventions[31] and the methods employed in the development of the HIV+D[14] and MANAS[15] mental health interventions and of the Health Activity Program (a behavioural activation based therapy)[32]. To address this general objective, 4 sub-studies will be undertaken, namely:

Sub-study 1: To adapt the HIV+D collaborative stepped care mental health intervention into primary health care in Mubende district to produce the Ebola+D mental health intervention

Sub-study 2: To adapt and translate the Problem Solving Therapy for Primary Care (PST-PC) treatment manual to the local rural situation in Mubende district

Sub-study 3: To evaluate the acceptability, feasibility and impact on mental health outcomes of the Ebola+D mental health intervention

Sub-study 4: To examine the costs of the Ebola + D mental health intervention

4.1.2. Study setting and context:

This component of the research will be undertaken in Mubende district. A range of stakeholders including mental health specialists (psychiatrists, clinical psychologists, social workers, psychiatric clinical officers and psychiatric nurses), health policy makers and implementers (MOH officials from the Mental Health and Substance Use Division, district health managers, managers of public health care facilities), primary health care workers (clinicians, general health

care workers), community members (members of the village health team, community leaders and service users) and Community Based Organisations (CBOs) involved in mental health will be involved in the local adaptation of the HIV+D mental health intervention to produce the Ebola+D mental health intervention. The feasibility, acceptability and impact on selected mental health outcome of the developed Ebola+D mental health intervention will be assessed over a 12 months' period.

4.1.3. Sub-studies:

Sub-study I: To adapt the HIV+D collaborative stepped care mental health intervention into primary health care in Mubende district to produce the Ebola+D mental health intervention

Study Lead: Mr Joshua Ssebunnya

Specific Objectives:

i) To adapt the HIV+D mental health intervention to the primary health situation in Mubende district in order to produce the Ebola+D mental health intervention

ii) To define the roles of each member of the Ebola+D mental health intervention team

iii) To develop a causal pathway of impact of the Ebola+D mental health intervention

iv) To identify possible mental health outcome indicators for the Ebola+D mental health intervention

Methods:

To undertake this, we shall employ the Theory of Change (ToC) based approach by De Silva and colleagues (2014)[33] to adapt the HIV+D mental health intervention to the post-Ebola primary health care situation in Mubende district in Uganda. This participatory methodology will achieve the following: i) increase the chances of success due to its sensitivity to the local context, stakeholder involvement and hence promotion of stakeholder buy-in; ii) describe the proposed

causal pathway to impact; iii) help identify barriers and strategies needed to implement the HIV+D mental health intervention in post-Ebola Mubende district so as to ensure full integration and the achievement of the ultimate goal of recovery from Ebola associated mental health problems (depression, anxiety and Post-traumatic stress disorder); and iv) identify possible outcome indicators of the intervention.

Theory of change (ToC) workshops with be held with the different categories of stakeholders who can influence the implementation of the intervention. To ensure that participants feel comfortable speaking out, separate ToC workshops will be held; the first workshop will be for mental health specialists, health policy makers and implementers and primary health care workers; and the second workshop will be for community members and CBOs involved in mental health. Each ToC workshop will begin with presentations highlighting the burden of common mental health problems in a post-Ebola rural community such as Mubende, the lack of mental health services in primary health care, the health system challenges of delivery of mental health services in primary health care and the need to integrate mental health services into primary health care. Through a process of participatory discussions, consensus will be sought on the programme's desired impact of having the integration of the management of common mental health problems in public health care facilities in Mubende district, Uganda. Each group will develop a programme theory describing how the intervention is expected to unfold and attain the desired impact. Also to be discussed are the different cadres who will deliver and support the different steps of care in the Ebola+D mental health integration model, how these will be implemented, the competencies required to perform these roles and responsibilities and how these will be acquired and maintained, and the monitoring of quality and fidelity of treatment.

A final workshop, bringing together all willing stakeholders will be conducted. Findings from the separate ToC workshops will be presented, and consensus will be made on the causal pathway to impact, the intervention and indicators, assumptions and rationale for each point along the causal pathway. The ToC workshops will be facilitated by an experienced ToC facilitator (Joshua Ssebunya, a clinical psychologist) who will construct the ToC maps. Co-facilitators appointed from the Ebola+D research team will take notes and audio record each meeting. Meetings will be held at MRC/UVRI & LSHTM Uganda Research Unit offices at Entebbe, and in Mubende district.

Such an approach has previously been used in the PRogram for Improving MEntal health care (PRIME)[32], a multi-country complex intervention aimed at generating evidence on how to integrate mental health into primary care through the development, implementation and evaluation of district level mental health care plans for priority disorders. More recently, this approach has been used to develop and pilot the HIV+D mental health intervention among adult persons living with HIV in Uganda[35].

Data management and analysis:

All data collected during ToC meetings will be managed according to the guidelines laid down by the Uganda Medical Research Council Unit. Data will be collected from a number of sources, including case notes and transcripts from the audio recordings of the ToC workshops, existing documentation from the MOH Department of Mental Health and Substance Use, minutes of the Ebola+D Research and Meetings with Mubende health officials. The ToC facilitator together with a research assistant will be responsible for compiling data into folders based on the subject matter. Emerging priority areas from each folder including recommendations will be noted and further explored through carrying out systematic reviews. Data will be categorised into themes based on content and the emerging themes will be shared with the Ebola+D research team. Consensus will be sought from the Ebola +D research group on issues such as outcomes, indicators, and interventions required to move from one point of the causal pathway to the next. The final output from this process will be a final ToC map that will specify the causal pathways, the required interventions, assumptions, indicators and rationale to attain the desired impact. These outputs will finally be shared with Ebola+D research collaborators and then used to further develop the Ebola+D mental health intervention.

Sub-study II: To adapt and translate the Problem Solving Therapy for Primary Care (PST-PC) treatment manual to the local rural situation in Mubende district

Study Lead: Christine Tusiime

Specific Objectives:

i) To adapt and translate the Problem Solving Therapy for Primary Care (PST-PC) treatment manual to the local rural situation in Mubende district

Background:

Problem Solving therapy (PST) is a low intensity cognitive-behavioral intervention that focuses on training in adaptive problem-solving attitudes and skills[36]. A meta-analysis by Bell and D'Zurilla (2009) using controlled outcome studies on efficacy of PST for reducing depressive symptomatology found that PST was equally effective as other psychosocial therapies and medication treatments and significantly more effective than no treatment[36]. Zhang and colleagues (2018) in a systematic review and meta-analysis of clinical trials examining PST for patients with depression and/or anxiety in primary care reported PST's effectiveness for primary care depression and/or anxiety[37]. Connolly and colleagues (2021) in a systematic review and meta-analysis to investigate the effectiveness of community-based mental health interventions by professionally trained, lay counsellors in low- and middle-income countries, observed that the use of professionally trained, lay counsellors to provide mental health interventions in low- and middle-income countries was associated with significant improvements in mental health symptoms across a range of settings[38].

Methods:

In this project we shall adapt the Problem Solving Therapy for Primary Care (PST-PC) treatment manual by Hegel and Areán (2011)[29] for use to train lay health workers (members of the Village Health Team) in the rural situation of Mubende. To locally adapt PST to the post-Ebola situation in rural Mubende district, we shall employ a methodology similar to that used by Chowdhary and others, (2016)[32] in India and most recently used by Kinyanda and colleagues (2020)[14] to adapt Behavioural Activation therapy to the local HIV care situation in Uganda.

The local adaptation process will be facilitated by Christine Tusiime a trained clinical psychologist with Butabika National Psychiatric Referral Hospital in Uganda (who also facilitated the local adaptation of Behavioural Activation therapy based HIV+D mental health intervention to the Ugandan HIV situation). The adaptation process will include holding

consultative meetings at the MRC/UVRI & LSHTM Uganda Research Unit offices in Entebbe with mental health specialists (psychiatrists, psychologists, social workers and psychiatric nurses) to review and adapt for use by lay health workers the PST-PC training manual. A second workshop will be held with potential community health workers (including members of the village health team, representatives of patient support groups and hospital ward attendants) at a venue in Mubende district. At this second workshop the draft locally adapted and translated PST-PC training manual will be used to train workshop participants. Later, workshop participants will be divided into groups to discuss the draft revised PST-PC training manual in terms of the following dimensions: i) feasibility (whether it is possible for community health workers, with appropriate training and supervision, to deliver this therapy); ii) acceptability (whether the therapy is regarded as suitable in the settings of Mubende public health care facilities); iii) effectiveness (whether the strategy brings about important and positive changes in depression and anxiety symptoms); iv) risk of harm (whether there is a potential for harm or risk involved in a community health worker delivering this strategy); and v) any suggestions for improving the delivery of the therapy. The discussion groups will then converge at a plenary session to share their findings.

Data management and analysis:

Presentations at the plenary sessions will be audio recorded and transcribed. Flip chart records from the different groups will also be collected. The resultant qualitative data will be analysed using thematic analysis techniques and used to improve the revised draft PST-PC training materials.

Translation of the PST-PC training manual:

Alongside the local adaptation of the PST-PC training manual, the Ebola+D research team will also undertake to systematically translate into Luganda (the dominant language of the study communities) the PST-PC manual so as to ensure content equivalence of the items. The translation process will involve the following steps: i) separate forward and back translation by teams of mental health professionals and lay people conversant with both English and Luganda; and ii) convene a consensus workshop of the translation teams to derive the versions with the most acceptable face validity.

<u>Sub-study III: To evaluate the acceptability, feasibility and impact on mental health outcomes of the Ebola+D mental health intervention</u>

Study Lead: Leticia Kyohangirwe

Specific Objectives:

i) To evaluate the acceptability and feasibility of the Ebola+D mental health intervention

ii) To assess the impact the Ebola+D mental health intervention on mental health outcomes

Background:

This sub-study will evaluate the acceptability and feasibility of the intervention and impact on mental health outcomes through implementation of the intervention in Mubende district. The Ebola+D mental health intervention will be implemented over 12 months at 11 public health care facilities (PHCFs; both health centre level IIs and IIIs) in Mubende district. At each of these PHCFs, 2 lay health workers (trained members of the village health team), 1 supervisor (an identified health worker from the PHCF), clinicians (from the PHCF) will form the core team that will deliver the Ebola+D mental health intervention; these will be supported by a mental health specialist (psychiatric nurse or psychiatric clinical officer from the Mental Health Department at Mubende Regional Referral Hospital). All these personnel will be assigned and trained into their expected roles and responsibilities and in the assessment of competency and, in the case of the psychological treatments, assessment of therapy quality[37].

At the PHCFs, community members accessing health services will be screened with the Self Report Questionnaire (SRQ-20), these found to meet eligibility criteria including having 'significant psychological distress' (SRQ-20 scores of ≥ 6)[27] will be triaged to the appropriate level of mental health treatment in the Ebola+D mental health intervention based on baseline SRQ-20 scores, suicidality risk assessment (a positive SRQ-20 item 16 ['do you feel that you are a worthless person?'] or/and item 17 ['has the thought of ending your life been in your mind?'], with a high suicide risk confirmed by the Supervisor), and response to treatment.

<u>Eligibility criteria for participating in this study:</u> i) community member of Mubende district staying within the catchment area of the PHCF; ii) 18 years and above, iv) able to communicate

in either English or Luganda (local language spoken in the study region and the language into which the questionnaires will be translated), v) has a WHO SRQ-20 score of 6 and above (except for Ebola survivors who are eligible even when their SRQ-20 score is either below or above 6).

Exclusion criteria: i) unable to engage with the research process for any reason that may include sensory impairment or cognitive impairment.

The enrolled respondents will be followed up until they have completed their therapy sessions and declared in remission. Monthly assessments will be undertaken to monitor their level of psychological distress using the SRQ-20 until psychological distress scores have fallen to less than 6. A patient will be declared in remission if after completing their prescribed therapy (psychoeducation, PST or/and medication) they remain symptom free (SRQ-20 scores of < 6) on two occasions one month apart. A full complement of supporting and resource materials including clinic posters, health worker aide-memoire, and patient and care-giver resource materials will be developed.

Data collection, management and analyses:

A complement of data collection tools to support this component of the study has been compiled together **(see Appendix I)**. Serial focus group discussions with lay health workers and in-depth interviews with supervisors and clinicians will be held. Data will be collected on the clinical process, including the engagement of patients, ease of implementing the decision support algorithm, barriers experienced in the delivery of the treatment and modifications made. Exit interviews will be held with a sample of both adherent and non-adherent (have dropped out of the intervention) respondents to describe their experiences and reasons for adherence or non-adherence. To assess effectiveness of the mental health intervention, SRQ-20 scores of study participants will be collected at baseline, 3 months and 12 months. Patients' satisfaction with Ebola+D mental health care will be assessed using a modified Patient Satisfaction Survey adapted from Ede and colleagues (2015)[40]. Participants' engagement with PST will be assessed after each session by the lay health worker using Stahl and colleagues (2017) Interventionists' Rating Scales of Participants' Engagement in PST[41]. This instrument has three scales: participation scale (one item), understanding of materials scale (one item), and homework effort scale (one item). The participation scale is rated on a Likert scale ranging from 1(none) to

6(excellent), the understanding of material scale from 1 (minimal understanding) to 3 (good understanding) and the homework effort scale from 1 (none) to 5 (excellent. Carers' (lay health workers, supervisors, mental health specialists) perceptions and satisfaction levels with integration efforts at their clinic will be assessed using a modified Staff Survey adapted from Ede and colleagues (2015)[40]. Monthly Health Management Information system (HMIS) records on mental health diagnoses of each of the participating PHCF clinic will be collected.

Summary of indicators that will be collected

Outcome	Definition	Source of Data	Endpoint
Improvement in psychological distress	Comparison of mean SRQ-20 scores at baseline, 3 months and 12 months	SRQ-20 scores	12 months
Fidelity of delivery of the intervention by lay health workers	Degree to which an intervention is implemented as described in the protocol.	Audio recording of lay health worker therapy sessions rated using a fidelity assessment scale	3 months during delivery of the therapy
Engagement with intervention by study participants	Participants' engagement with PST, undertaken after each session, assessment done by lay health worker on three scales: participation scale (one item), understanding of materials scale (one item), and homework effort scale (one item). Each of these scales is rated on a Likert scale ranging from 1(none) to 6(excellent)	Stahl et al., (2017)'s Interventionists' Rating Scales of Participants' Engagement in PST[41]	During the administration of the therapy in first 3 months
Engagement with intervention by study participants	Proportion of persons with psychological distress who receive recommended number of treatment sessions	Study records of each patient	3 months
Patients' satisfaction with Ebola+D mental health care	To be assessed using a modified Patient Satisfaction Survey adapted from Ede and colleagues (2015)[38]	Modified Patient Satisfaction Survey adapted from Ede and colleagues (2015)[40]	3 months
Carers' perceptions and satisfaction levels with integration efforts at their PHCF	Lay health workers, supervisors, clinicians, mental health specialists perceptions and satisfaction levels with integration efforts at their clinic	Assessed using the modified Staff Survey adapted from Ede and colleagues (2015)[40]	3 months
Uptake of mental health services	Number of patients at PHCF who are accessing mental health services	Monthly HMIS records of each participating PHCF	12 months

Outcome	Definition	Source of Data	Endpoint
Functional impairment	Degree of functional impairment in the domains of: work/school, social life and home life or family responsibilities	Assessed using the Sheehan Disability Scale (SDS)[42]	12 months

Qualitative data will be compiled and analysed using thematic analysis techniques while process indicators will be collected and analysed using descriptive statistics. Data collection and analysis will be undertaken iteratively and used to improve the intervention in 3 monthly cycles. Preliminary data on the effectiveness of the intervention will be obtained by comparing mean SRQ-20 scores at baseline with those at 3 months.

Sub-study IV: Sub-study to examine the costs of the Ebola + D mental health intervention

Study Lead: Barbra Kiconco

Specific Objectives:

i) To examine the costs of the Ebola +D intervention from the providers' perspective

Background:

This sub-study will examine the costs of the Ebola +D intervention from the providers' perspective. This perspective will enable us to determine how much it will cost for the health system to set up and operate the intervention at the health facility level. We will estimate the average total unit cost per patient for screening and treatment of mental health disorders at the health facilities in Mubende district.

Methods:

A combination of retrospective and prospective methods of cost data collection will be used. Both macro (top-down) and micro (bottom-up) costing approaches will be applied. The disaggregated provider costs will be summed to produce a unit cost per patient for screening and treatment of mental health problems from the provider perspective.

Data Collection

The data collection team will consist of a health economist and a health economics research assistant. Cost data collection on the Ebola+D intervention is expected to start on the 2nd May 2023 over a 12 months' period, using a standardized Excel based cost data collection tool that we will develop. Published literature will be scanned to identify the cost categories used by similar previous studies when developing the tool. In addition to this, we will review the Ebola +D intervention documents to develop a cost category list for the program. The developed tool will be piloted in two Ebola+D facilities (one Health Centre II, one Health Centre III) and tool modifications will be made before costing the remaining 9 facilities.

Resources/ Inputs

To determine the costs from the providers' perspective, the value and quantity of resources/ inputs required to produce outputs like outpatient visits for screening, treatment and follow-up are required. These inputs will be categorized into three, start-up costs, personnel costs and operational costs as indicated in the Table 1. Start-up costs include the costs of activities and equipment needed when setting up the intervention (these are a one-off expenditure). Personnel category includes the cost of all staff involved in the Ebola+D team while the operational cost category includes the cost of all items that are related to operational activity of the intervention.

Table 1: Cost Categories, Information to be obtained and data sources for the cost analysis of the Ebola+D Intervention

**Cost Category**	**Information to be obtained**	**Data Source**
**A. START-UP COSTS**		
**Health Worker Training**<br>Training costs per participant (Lay health workers, clinician and mental health specialist)	-Duration of training (number of days)<br>-Number of health workers trained and their cadre<br>-Transport costs to attend trainings<br>-Accommodation costs<br>-Training materials such as therapy manuals<br>-Costs of hiring training venue<br>-Costs of supplies for training such as pens, notebooks, food<br>-Number of refresher trainings<br>-Costs of trainers (if applicable)<br>-Hiring costs (if applicable)	Project Training Records

**Furniture and Equipment**	Costs of tables, chairs, metallic cabins and umbrellas that will be delivered to the various health facilities.	-Project Records<br>-MRC procurement records
**B. PERSONNEL COSTS**		
**Service delivery costs**<br>Cost for each service delivered by the different health workers involved in the study such as trained lay health workers, clinicians and specialized mental health specialists, phlebotomists	-How many health talks were given?<br>-How many people attended the health talks?<br>-How many patients were screened?<br>-How many patients were enrolled in the study?<br>-How many patients are receiving psychoeducation?<br>-How many patients are receiving problem-solving therapy?<br>-How many times do they review patients receiving treatment?<br>-What is the average time spent delivering treatment to patients? (number of minutes/hours)<br>-How many mental health distress related inpatient bed days were recorded at the facility?<br>-How many patients were suicidal?<br>-How many patients were receiving SSRIs?	-Time study<br>-Facility Data Managers<br>-Intervention Data
**C. OPERATIONAL COSTS**		
**Capital- Building Space**<br>(Costs for space utilized by the Ebola+D intervention)	-Where are health talks given?<br>-Where are the patients screened, enrolled and treated from?<br>-Where are the tests conducted?<br>-How many square meters are the facility?<br>-What is the value of the facility?<br>-What are the maintenance costs ?	Direct measurements of building space utilized by the intervention
**Capital – Equipment**<br>(Costs of Equipment utilized by the Ebola+D intervention)	-What equipment are used for the tests carried out?<br>-What is the price of the equipment used?<br>-What are the maintenance costs for the equipment?	-Facility Accountant<br>-Facility Administration records
**Drugs**<br>(Costs of recurrent drugs used in the intervention)	-What drugs are used to manage mental health problems?<br>-What is the origin (producer)<br>-What is the dose and strength of the drug?<br>-What is the mode of administration?<br>-What is the cost of the drug?	National Medical Stores price list
**Supplies**<br>(Costs of recurrent supplies used in the intervention)	-What supplies are used for tests (such as pregnancy tests)?<br>-What supplies are used for screening, enrolment, treatment?	-MRC Project Records<br>-Health Facility Records<br>-National Medical Stores price lists.

	-What are the costs of these supplies?	
**Utilities**<br>(Cost of overhead costs)	What are the recurrent expenditures for electricity, water, internet, telephone / airtime for the most recent financial year?	Facility Accounts records

Direct Costs

Direct medical costs of treatment will be disaggregated and descriptively analysed from baseline to endline. Categories of cost-related data will be assessed to determine the major cost drivers for delivering mental health services for EVD affected communities.

Research and Start-up Costs

All research costs and start-up costs will be reported separately from the cost of delivering the intervention.

Provider Cost Measurement and Valuation:

The quantity of resources used and their respective unit costs will be used to calculate the unit cost for each service output such as, outpatient visits (including screening, consultation, treatment and follow-up visits), inpatient bed days and medication. The function below explains the calculation of the service output unit cost.

$OUC=n(R) \times p(R)$

Where OUC is the unit cost per service output, n(R) is the quantity of resources used and p(R) is the unit cost of resources used.

The total unit cost per patient will be calculated by multiplying the quantity of each service output by the unit cost of the service output from screening to the end of treatment for each patient in the study. The function below explains the calculation of the total unit cost per patient.

$TUC=q(S) \times OUC$

Where TUC is the total unit cost per patient, q(S) is the quantity of each service output and OUC is the unit cost per service output.

The average cost per patient for screening and treatment will be derived and compared across the 11 facilities using a t-test.

Time Study

In addition to the data that will be collected on personnel salary and benefits from the facility records, we will conduct a time study to capture the proportion of each staffs' time allocated to the intervention[43,44]. We shall develop an activity log with a list of duties such as delivering health talks, delivering psychoeducation, problem solving therapy and drug prescription that will be prospectively completed by intervention staff to record their time spent on Ebola+D related activities. Health workers involved in the study will be trained to fill in the activity log. This data will be collected at specific sample time periods across the study (for example 1 week during baseline and 3 months) to reduce the additional research burden to staff that are already working at capacity.

Sensitivity Analysis and Uncertainty

We shall carry out a deterministic sensitivity analysis to deal with uncertainty and examine the robustness of the Ebola+D intervention costs at the extreme points[45]. We shall use the scenario analysis where we will calculate program costs using two scenarios, the most expensive and least expensive case to see how cost of the program changes. We shall first identify the major cost components of the intervention that are likely to change overtime and vary across different settings. For example, for the Ebola+D intervention, personnel costs such as salaries of the lay health workers will be varied with highest and lowest salary level.

Risk Assessment and Management

Untreated common mental disorders of depression, anxiety and post-traumatic stress disorder when untreated will negatively affect the mental wellbeing of patients including negatively affecting their quality of life, their social and work/academic functioning and may predispose them to negative behaviours such as alcohol and substance use, risky sexual behaviour and suicidal behaviour. It is therefore important that clinically significant common mental health problems are diagnosed and treated. There are no known side effects of psychological treatments including PST that will be used in this project. There are however important adverse effects that could arise from taking selective serotonin re-uptake inhibitors (SSRIs) such as

fluoxetine[46] and drug to drug interactions between the antidepressants and other treatments for chronic illnesses such as antiretroviral therapy for HIV/AIDS[47]. The clinicians and the mental health specialists' health worker who will participate in this study will prescribe the SSRIs medication after they have been taken through an orientation training to update them on these potential adverse events.

Due to safety concerns (although the evidence is still ambiguous) about the use of fluoxetine (an SSRI) in pregnant women[48,49], we shall be guided by the following: i) All women enrolled into this study who require an SSRI will have to undertake a pregnancy test, and if found to be positive shall only be offered the option of psychotherapy (PST); ii) Pregnant non-responders to PST will be referred directly to specialist mental health workers (who will treat them according to national guidelines); iii) the study will follow the same procedure for nursing mothers (lactating mothers); iv) Women on antidepressant medication who become pregnant during the course of the study will be offered a choice to withdraw from the antidepressant with an explanation of the risks and benefits and, if needed, offered PST.

4.2 SUB-COMPONENT II: TO INVESTIGATE THE NATURE OF MENTAL HEALTH PROBLEMS OF COMMUNITY MEMBERS ACCESSING THE EBOLA+D MENTAL HEALTH INTERVENTION AND FACTORS ASSOCIATED WITH TREATMENT OUTCOMES (EBOLA+D COHORT SUB-COMPONENT)

4.2.1 General Objectives:

To address the general objective of investigating the nature of mental health problems of community members accessing the Ebola+D mental health intervention and social, psychological and immunological factors associated with treatment outcomes (Remission at 3 months and Recovery at 12 months). This research sub-component will be undertaken in two sub-studies:

Sub-study V: To investigate the nature of mental health problems of community members accessing the Ebola+D mental health intervention and factors associated with treatment outcomes

Sub-study VI: Immunology sub-study

4.2.2 Sub-studies:

Sub-study V: To investigate the nature of mental health problems of community members accessing the Ebola+D mental health intervention and factors associated with treatment outcomes

Study Lead: Richard Mpango

Specific Objectives:

i) To investigate the nature of mental health problems of community members accessing the Ebola+D mental health intervention

ii) To investigate the social and psychological factors associated with Ebola+D mental health treatment outcomes

Study design:

A cohort of EVD affected individuals accessing the Ebola +D mental health intervention will be recruited and followed up for 12 months with three assessments baseline, 3 months and 12 months. The study participants in this cohort will include about 1,000 respondents who will include: patients/survivors of EVD, suspected cases of EVD, family members of EVD survivors, grieving family members who have lost loved ones to EVD, community members who have been negatively impacted by EVD in anyway, health workers and volunteers. At each of the three study visits (baseline, 3-months and 12-months), these study participants will be assessed by trained research assistants from the Mental Health Section of MRC/UVRI & LSHTM Unit (experienced research assistant consisting of psychiatric nurses and psychiatric clinical officers) using a locally translated structured psychiatric assessment protocol that has previously been used by the Mental Health Section[50-52].

Eligibility criteria for study participants: i) community member, health care workers and Ebola volunteer workers in Mubende district accessing the Ebola+D mental health intervention; ii) 18 years and above, iv) able to communicate in either English or Luganda (local language spoken in the study region and the language into which the questionnaires will be translated), v) consented to participate in the study.

Exclusion criteria: i) unable to engage with the research process for any reason that may include sensory impairment or cognitive impairment.

Data Collection Procedure:

A structured interview will be administered to recruited study respondents using a standardised questionnaire (**see Appendix II**). The interview questionnaire will initially be piloted among 20 study participants.

Data Collection tools

The data collection tool for this study will consist of a standardised structured interviewer administered questionnaire consisting of various modules that have previously been used by the Mental Health Section[50-52]. Study modules that will be used for the first time in this socio-cultural context will be taken through a local adaptation process that will consist of: i) separate forward and back translation by teams of mental health professionals conversant with both English and the local language of translation; ii) convene a consensus workshop of the translation teams to derive the versions with the most acceptable face validity; and iii) testing the module with study participants at a pilot study in order to enhance clarity.

The nature of mental health problems and neurocognitive impairment among community participants accessing the Ebola+D intervention will be determined. We shall also determine the social, psychological and biological (including immunological and neurodegenerative) markers of mental health treatment outcomes. An understanding and the development in the African setting of social, psychological and biological markers of mental health treatment outcomes is important and a prerequisite for the participation of African health systems in the coming new revolution of artificial intelligence (AI) aided personalised medicine. The social, psychological and biological variables that are going to be explored in this study as potential markers of mental health treatment outcomes have previously been associated with prevalent and incident mental illness in the Ugandan setting by the Mental Health Section[50-53]. The variables that will be collected as part of this study will be grouped as follows: socio-demographic factors, mental health and neurocognitive factors, stress factors, vulnerability/protective factors, work related factors, impairment factors and mental health treatment outcome measures.

i) Socio-demographic factors

A questionnaire coded on a tablet will be used to collect data on the following: a) study site; b) sex; c) age; d) religion; e) highest educational level attained; f) marital status; g) employment status (type of employment contract; h) living arrangement; i) socio-economic index (which will be constructed from a list of common household items[54]; and j) status of study participants with regards to the Ebola epidemic: patients/survivors of EVD, suspected case of EVD, family members of EVD patient/survivors, community members who lost a loved one to EVD, other community members, health worker, and community worker.

ii) Mental health problems

Psychiatric diagnoses/problems of major depressive disorder, generalised anxiety disorder, alcohol misuse, use of other substances, suicidality, insomnia, post-traumatic stress disorder and psychosomatic complaints will be assessed in this study.

Major depressive disorder

Major Depressive Disorder in this study will be assessed using the Patient Health Questionnaire (PHQ-9), a DSM based diagnostic and symptom severity measure that has been used extensively in Africa including validation[55-56]. The PHQ-9 consists of 9 depression symptom items which are scored on a Likert scale of: 0= ‘not at all’; 1= ‘several days’; 2= ‘more than half the days’; 3= ‘nearly every day’. Interpretation of PHQ-9 scores: 1-4: this is considered minimal depression, which suggests that the respondent may not need depression treatment; 5-9: this is considered mild depression; 10-14: this is considered moderate depression; 15-19: this is considered moderately severe depression; 20-27: this is considered severe depression. The PHQ-9 has a 10th item which assesses degree of distress or impairment as a result of the depressive symptoms. The 10th item reads as follows: ‘If you checked off any problem on this questionnaire so far, how difficult have these problems made it for you to do your work, take care of things at home, or get along with other people?’, with possible responses being: 1= ‘not difficult at all’; 2= ‘somewhat difficult’; 3= ‘very difficult’; and 4= ‘extremely difficult’.

Generalised anxiety disorder

Generalised anxiety disorder in this study will be assessed using the Generalized Anxiety Disorder 7 (GAD-7), a DSM based self-reported questionnaire for screening, diagnosis and severity measuring of generalised anxiety disorder[57]. Each item asks the individual to rate the severity of his or her symptoms over the past two weeks with possible responses being: 0= 'not at all'; 1= 'several days'; 2= 'more than half the days'; and 3= 'nearly every day'. GAD-7 total score for the seven items ranges from 0 to 21. Scores of 5, 10, and 15 represent cut-points for mild, moderate, and severe anxiety, respectively.

Alcohol misuse

Alcohol misuse in this study will be assessed using the Alcohol Use Disorders Identification Test (AUDIT)[58]. The AUDIT is a 10-item screening tool developed by the World Health Organization (WHO) to assess alcohol consumption, drinking behaviours, and alcohol-related problems. The clinician-administered version of the AUDIT will be used in this study. A score of 8 or more is considered to indicate hazardous or harmful alcohol use. The AUDIT has been validated across genders and in a wide range of racial/ethnic groups and is well suited for use in primary care settings.

Use of drugs of abuse

This will be assessed by asking respondents whether they use any of the following substances of abuse: marijuana, Khat, Kubab, cocaine and heroin.

Use of Tobacco

This will be assessed by asking respondents whether they regularly use tobacco products including through smoking, sniffing and chewing.

Suicidality

Suicidality will be assessed using the suicidality module in the Diagnostic and Statistical Manual of Mental Disorders (DSM) based structured interview, the M.I.N.I. neuropsychiatric interview (MINI Plus)[59]. 'Moderate to high risk for suicidality' is defined as a score of 9 and above in this suicidality module.

Insomnia

Insomnia will be assessed using the Insomnia Severity Index (ISI), which consists of 7-items which are each scored on a Likert scale where: 0= 'none'; 1= 'mild'; 2= 'moderate'; 3= 'severe'; 4= 'very severe'[60]. The scores of the 7 items are summed together and interpreted as follows: 0–7 = no clinically significant insomnia; 8–14 = sub-threshold insomnia; 15–21 = clinical insomnia (moderate severity); 22–28 = clinical insomnia (severe).

Psychosomatic complaints

Somatic symptoms in this study will be assessed using the 8-item Somatic Symptom Scale (SSS-8)[61], a brief, patient-reported outcome measure of somatic symptom burden. The items of the SSS-8 scale are scored on a 5-point Likert scale where 0=not at all', 1= 'a little bit', 2= 'somewhat', 3= 'quite a bit' and 3= 'very much'. The total scores on the SSS-8 will be categorised as 'no to minimal' (0-3 points), 'low' (4-7 points), 'medium' (8-11 points), 'high' (12-15 points), and 'very high' (16-32 points) somatic symptom burden[61].

Post-Traumatic Stress Disorder

Post-Traumatic Stress Disorder (PTSD) will be assessed in this study using the PTSD Checklist for DSM-5 (PCL-5)[62]. The PCL-5 is a 20-item self-report measure that assesses the 20 DSM-5 symptoms of PTSD. The PCL-5 inquires whether in the past month an individual has been bothered by a list of 20 symptoms that are scored on 5-point Likert scale ranging from: 0= 'not at all'; 1= 'a little bit'; 2= 'moderately'; 3= 'quite a bit'; 4= 'extremely'. A total symptom severity score (range - 0-80) can be obtained by summing the scores for each of the 20 items. DSM-5 symptom cluster severity scores can be obtained by summing the scores for the items within a given cluster, i.e., cluster B (items 1-5), cluster C (items 6-7), cluster D (items 8-14), and cluster E (items 15-20).

A provisional PTSD diagnosis can be made by treating each item rated as 2 = 'moderately' or higher as a symptom endorsed, then following the DSM-5 diagnostic rule which requires at least: 1 B item (questions 1-5), 1 C item (questions 6-7), 2 D items (questions 8-14), 2 E items (questions 15-20).

(iii) Neurocognitive impairment

Neurocognitive impairment will be assessed among EVD patients/survivors and matched controls (matched on age, sex and highest number of years of formal education attained) in a ratio of 1:1. The neurocognitive battery that has previously been used by the Section, will include: a) Symbol Digit Modalities Test (attention, visual scanning, motor and psychomotor speed); b) Trial Making Test Part A (speed of information of processing); c) Trial Making Test Part B (executive function); d) Grooved Pegboard Test (motor function); e) Timed finger tapping (from the International HIV Dementia Scale- IHDS, for motor speed); e) Timed alternating hand sequence test (from the IHDS for psychomotor speed); and Recall of four items at two minutes from the IHDS (memory)[63-65].

iv) Stress factors

The following stress factors will be assessed in this study: Ebola knowledge, sentiments and risk perception and personal stressors.

Ebola Knowledge, Sentiments and Risk Perception

Ebola knowledge, sentiments and risk perception will be assessed using the modified Assessment of Public Knowledge, Attitudes, and Practices Related to Ebola Virus Disease Prevention and Treatment in Uganda, 2019 Questionnaire by Musaazi and colleagues (2022)[66]. The modules from that questionnaire that will be used in this study will include: Ebola Knowledge (12-items), Ebola Sentiments (5-items) and Ebola Risk Perception (3-items). This questionnaire was developed and has previously been used in Uganda[66].

Personal stressors

Personal stressors will be assessed using the negative life events module of the Uganda modified European Parasuicide Interview Schedule[67-68]. For this study respondents will be required to report whether they had experienced negative life events in the last 6 months. The negative life events that will be considered in this study will examine stressors such physical illness, interpersonal conflict and bereavement in relation to the individual or significant others (spouse, children, parent or sibling). Study participants will be required to state whether they had experienced an event or not.

v) Vulnerability and Protective factors

The following factors could serve as either vulnerability/protective factors of the relationship between stress and mental health problems, these are: Maladaptive Coping Style, Resilience and Social Support.

Maladaptive Coping Style

Coping style will be assessed using the Carver Brief COPE[69] which is a 28-item measure comprising 14 scales: active coping, planning, positive reframing, acceptance, humor, turning to religion, venting of emotions, mental disengagement, denial, substance use, behavioural disengagement, and emotional support. Each scale consists of two items. For each item, study participants will be asked to respond on a 4- point Likert-scale: 1= ‘I did not do this at all’; 2 = ‘I've been doing this a little bit’; 3 = ‘I've been doing this a medium amount’; and 4 = ‘I've been doing this a lot’ to the activities in the past 3 months. This instrument has previously demonstrated good internal consistency in the Ugandan socio-cultural environment[50-52].

Resilience

Resilience in this study will be assessed using the Connor-Davidson Resilience Scale (CD-RISC)[70]. The Connor-Davidson Resilience scale (CD-RISC) comprises of 25 items, each rated on a 5-point scale where 0= ‘not true at all’; 1= ‘rarely true’; 2= ‘sometimes true’; 3= ‘often true’; 4= ‘true nearly all the time’. Higher scores reflect greater resilience. This instrument has previously demonstrated good internal consistency in the Ugandan socio-cultural environment[50-52].

Social support

Social support in this study will be assessed using the Multidimensional Scale of Perceived Social Support (MSPSS)[71]. The MSPSS is a 12-item instrument that was designed to assess perceptions about support from family, friends and significant others. Each item on this scale will be graded according to a 7 point Likert Scale where 1= ‘if you very strongly disagree’; 2= ‘if you strongly disagree’; 3= ‘if you mildly disagree’; 4= ‘if you are neutral’; 5= ‘if you mildly agree’; 6= ‘if you strongly agree’; 7= ‘if you very strongly agree’. This instrument has previously demonstrated good internal consistency in the Ugandan socio-cultural environment[50-52].

vii) Work factors

Job satisfaction

Job satisfaction will be assessed using the Hackman and Oldham's (1975)[78] job satisfaction scale. This scale measures job satisfaction in terms of extrinsic satisfaction (9 question items) and intrinsic satisfaction (11 question items) on a 5-point Likert scale from very satisfied (5) to very dissatisfied (1). This scale has previously been shown to have satisfactory internal consistency in the Ugandan socio-cultural environment.

Work environment

A healthy work environment is a critical factor in health worker satisfaction, retention, and patient outcomes. Work environment in this study will be assessed using the short 10-item scale based on the Practice Environment Scale of the Nurse Work Index[79]. This instrument has questions such as, 'Nurses at the health centre have opportunities to participate in decisions that affect centre policies'. The questions in this tool will be modified so that they are generalisable to all cadre of health workers, for example the above question will now read, 'health workers at the health centre have opportunities to participate in decisions that affect centre policies'. These questions are scored on a 4- point Likert scale where: 1= 'strongly disagree', 2= 'disagree', 3= 'agree', and 4= 'strongly agree'.

viii) Impairment

Degree of disability

Degree of functional impairment in this study will be assessed using the Sheehan Disability Scale (SDS)[42], that assesses degree of impairment in the 3 domains of: work/school, social life and home life or family responsibilities. On this scale, the respondent rates the extent to which work/school, social life and home life or family life responsibilities are impaired by his or her psychiatric symptoms on a 10-point visual analog scale. The 10-point visual analog scale uses spatiovisual, numeric and verbal description anchors to simultaneous assess disability. The three domains can also be summed into a single dimensional measure of global functional impairment that ranges from 0 (unimpaired) to 30 (highly impaired). There is no recommended cut off score, change-over-time in scores can be used to monitor response to treatment. Scores of 5 or greater on any of the three dimension denotes significant functional impairment.

ix) Mental Health Treatment Outcomes

The following mental health treatment outcomes be defined:

a) ***Remission at 3 months:*** After completion of prescribed mental health treatment step(s), having SRQ-20 scores < 6 on two occasions 4 weeks apart.

b) ***Recovery at 12 months:*** After attaining remission at any point during the study, if SRQ-20 scores < 6 at 12 months.

c) ***Relapse at 6 or/and at 12 months:*** After attaining remission, if at either 6 months or/and at 12 months the SRQ-20 scores rise up again to 6 or more.

Table 2: Study Variables by Status of Study Participants

Study Variables	PSE	FMS	CLL	OCM	HW	CW
Socio-demographic factors	√	√	√	√	√	√
Mental health problems	√	√	√	√	√	√
Neurocognitive impairment	√					
Ebola risk perception	√	√	√	√	√	√
Personal stressors	√	√	√	√	√	√
Maladaptive coping style	√	√	√	√	√	√
Resilience	√	√	√	√	√	√
Social support	√	√	√	√	√	√
Job satisfaction					√	
Work environment					√	
Immunological factors	√	√	√	√	√	√
Degree of Disability	√	√	√	√	√	√
Mental Health Treatment Outcomes	√	√	√	√	√	√

Note: Status of study participants with regards to the Ebola epidemic: Patients/Survivors of EVD (PSE), Family Members of EVD Patient/Survivor (FMS), Community members who lost a loved one to EVD (CLL), Other Community Members (OCM), Health worker (HW) and Community Worker (CW)

Data Management:

At the public health care facilities, study data will be electronically entered on tablets into a GCP-compliant, mobile application [Research Electronic Data Capture (REDCap), Nashville, TN, USA]. REDCap has a built-in audit trail that automatically logs activity including log-ins, user

rights, data entry/modification, data export, and running reports. Only the PI, the trial coordinator, the data manager and the Statistician will have access to this database.

Data from tablets, which will be partially anonymised (participant names excluded), will be synced once daily over a secure connection with a web-based REDCap database. The system will be centrally hosted at the MRC/UVRI & LSHTM data centre.

The data manager to identify data that are missing, inconsistent, or out-of-range, will run internally developed Stata verification /cleaning do-files. The clinic team will perform clinical quality checks to identify potential errors not captured in the automated verification process.

Query resolution

The trial coordinator or data managers will raise queries through the REDCap query management system and assign it to a member of the study team at the respective site, who will respond to it. Only after a satisfactory response is given will the query be closed. Pending queries will be allowed up to 2 days if the patient is still hospitalised or 5 days if patient has been discharged.

Statistical Analyses:

Data will be checked for missingness and sparsity for all the variables. Demographic and psychosocial characteristics of the participants will be summarised using frequencies and percentages for categorical variables, means and medians will be used to summarise continuous variables depending on the nature of the distribution by the different types of disorders. We will assess the factors associated with Remission at 3 months, Relapse at 6 or/ and 12 months and Recovery at 12 months using random effects logistic regression models. *A priori* factors to consider will include gender, education level, age, and baseline severity in addition to the factors that will be identified to be associated with the outcome from the univariable analysis.

Using machine learning approaches, we shall explore social, psychological and biological (immunological) markers of response to therapy type in the Ebola+D mental health intervention. Ebola+D mental health intervention constitutes of three therapy types of increasing intensity, namely, psychoeducation, problem solving therapy (PST) and medication with the Selective Serotonin Reuptake Inhibitor, Fluoxetine given in a stepped care model. Using stepped care approaches such as that suggested in Ebola+D mental health intervention may take up to three months before a patient can get therapy that will induce remission of their mental health disorder.

Treatment stratification has been suggested as one approach to reduce this time interval. Using unsupervised machine learning algorithms, we intend to identify the different groups of patients (clusters) that may exist within our cohort in relation to response to the different types of therapy. This is intended to inform our future work that will be focused on further development of a stratified mental health care model. We will then identify the patient level characteristics as well as biological markers linked to response to the different therapy types. These analyses will be completed using STATA 17 and Python software's.

Sub-study VI: Immunological markers sub-study
Study Lead: Andrew Obuku

Specific Objectives:
i) To investigate the immunological factors associated with EVD associated mental health problems
ii) To investigate the immunological factors associated with Ebola+D mental health treatment outcomes

Background:
Ebola causes a pathologic inflammatory cytokine storm evidenced by the secretion of pro- and anti-inflammatory cytokines, chemokines, soluble receptors and growth factors/regulators[72]. This state of hyperinflammation, may disrupt the function of the Blood Brain barrier (BBB) by increasing BBB's permeability and trigger an onset of neurological symptoms. Indeed, Ebola RNA has been detected in the cerebrospinal fluid (CSF), suggesting that Ebola crosses the BBB[73]. There is growing evidence for possible central nervous system (CNS) viral invasion including clinical and imaging features suggestive of meningoencephalitis and meningitis[8,9,12,13]. Psychiatric disorders such as depression, anxiety and PTSD have been associated with hyperinflammation including studies by the Mental Health Section[53,74]. Cytokines, growth factors, chemokines and soluble receptors have been implicated in neuroinflammation leading to cognitive deficits[75,76]. However, there is paucity of knowledge on how the immune system including the CNS responds during psychiatric treatment. The objective is to profile the global pathophysiological processes of depression, anxiety, PTSD and neurocognitive impairment among patients affected and infected by EVD Sudan outbreak of 2022 in Uganda and associate these with treatment outcomes.

Methods:

From all participants, twelve millilitres (mls) of venous blood will be collected in 10 ml EDTA tube and 2.5mls Pax gene tube at baseline only. For Ebola survivors blood will be drawn at three time points (baseline,3 months and 12 months). The EDTA blood will be centrifuged, plasma & packed cells stored in cryovials in -20°C freezer. The paxgene tubes will be kept on racks at -80°C within 3 hours of blood draw.

The plasma will be and tested for cytokines, chemokines, soluble receptors & growth factors such as IL-1RA, IL-1β, MCP-1, TNF-α, GM-CSF, IL-8, IL-6, IL-10, APRIL, bNGF, IL-23, Fractalkine, Eotaxin, VEGF-A and neurodegeneration markers such as Amyloid β (1-40), Amyloid β (1-42), FGF-21, Kallikrein-6 (KLK-6), NCAM-1, Neurogranin (NRGN), Tau (total), Tau (pT181), TDP-43, Apolipoprotein E4, Clusterin (Apo J), Complement Factor H, and Fetuin-A using the Luminex Technology[77].

Metabolomics can assist with the discovery of disease specific biomarkers. This can lead to prediction, detection and diagnosis of psychiatric illnesses. The metabolites detected include amino acids, alcohols, vitamins, ATP, ADP and monosaccharides. We propose to use global discovery non targeted metabolomics approach in the analysis since there are no targeted panels established for psychiatric illnesses. Metabolites will be extracted from EDTA plasma and separated using liquid chromatography and detected using mass spectrometry.

RNA-Seq will be performed using the HiSeq 4000 (Illumina Inc.) which can generate ~600 Gb of sequence for a paired-end 100 base read run. SMARTer Ultra Low RNA Kits will be used to generate libraries from low amounts of total RNA. The quality of the libraries will be verified using DNA-1000 Kits (Agilent) and quantified using the Qubit 2.0 Fluometer (www.invitrogen.com). Alignment will be performed using the rapid Spliced Transcripts Alignment to a Reference (STAR) Aligner. Custom annotation tracks include ENSEMBL transcripts, known noncoding RNAs (ncRNA) and putative novel ncRNAs. Normalization and differential transcript analysis will be conducted using the EdgeR framework with False Discovery Rate adjustment.

Linear regression models will be used to regress gene expression against psychiatric illness to identify transcriptional profiles and biochemical mechanisms that led to recovery of each particular

illness. The integration of these results (cytokines, chemokines, soluble receptors, transcripts and metabolites) will lead to the development of models that can define the mechanisms associated to depression, anxiety and PTSD. These findings could lead to the establishment of novel clinical interventions pathways and personalised medicine.

4.3 SUB-COMPONENT III: TO EXPLORE THE EVD ASSOCIATED NEGATIVE BELIEFS AND LIVED OUT EXPERIENCES OF AFFECTED MEMBERS OF THE COMMUNITY (EBOLA+D QUALITATITIVE SUB-COMPONENT)

Study Lead: Rwamahe Rutakumwa

This sub-component will be undertaken to answer the questions: i) What is the lived experience (including psychological distress, psychosocial challenges) of the different categories of persons affected by the EVD (patients/survivors of EVD, suspected cases, family members, contacts of EVD and health workers and volunteers)? and ii) What are community beliefs (including misperceptions such as witchcraft) and coping mechanism employed by the community towards the threat of Ebola?

Study Design:

A qualitative study using a phenomenological research design will be undertaken over a period of 12 months in Mubende District, the EVD epicentre, to explore the lived experiences of various categories of persons affected by EVD, and how they are coping before and after receiving psychosocial support interventions. The study will draw participants from all the nine sub-counties constituting Mubende District. The participants will include (i) patients/survivors, (ii) suspected cases who were kept in isolation, (ii) family members of survivors and those who lost lives, (iii) community members [including traditional healers and opinion leaders], (iv) health workers and (iv) volunteers.

Methods:

The different categories of participants will be purposively sampled, and further stratified for gender balance especially among EVD survivors and their family members. For these two categories of participants, the purposive sample will be based primarily on the records from EVD survivors' clinics. These clinics will contact the EVD survivors and their family members to obtain their permission to share their contacts before these can be availed to the study team. Prospective participants will then be traced with the help of VHTs that are spread across the nine sub-counties of Mubende District. For the health workers and volunteers, these will be approached at their facilities.

Serial in-depth interviews will be conducted with up to 15 EVD patients/survivors, 5 suspected cases who were kept in isolation, 15 family members, 8 community members, 8 health workers and 8 volunteers, with the first set of interviews being held at baseline/beginning of the study. The first set of interviews will mainly elicit participants' perspectives on their lived experiences with EVD and how they have coped, and explore their psychosocial support needs. The interview questions at this stage will revolve around (a) participants' and community beliefs about EVD and their influence on health seeking behaviour or treatment choices, (b) perspectives on being treated or working at the EVD Treatment Unit, or being held in isolation on being suspected to have contracted EVD, (c) psychosocial challenges and distress of being affected by EVD and how they have coped, and (d) their psychosocial support needs. The participants will then be followed up at Months 3 and 12 of the study to collect qualitative data on their experiences and coping after receiving specific psychosocial support interventions.

Data collection will be conducted at the health facility/EVD Treatment Unit or at a location preferred by the participant. With the permission of the participant, the interviews will be voice-recorded and subsequently transcribed verbatim. An interpretative phenomenological analysis of data will be done.

5.0 ETHICAL CONSIDERATIONS, MONITORING AND AUDITING

The study will obtain ethical approval from the Uganda Virus Research Institute's Research Ethics Committee, the London School of Hygiene and Tropical Medicine Research Ethics

Committee and the Uganda National Council for Science and Technology. Study participants will be invited to consent after being provided with adequate information about the study. Respondents found to have significant psychiatric problems will be referred to the nearest health facility for treatment.

The Sponsor will undertake both remote and onsite monitoring arrangements to monitor the study. Auditing by the Sponsor and inspection by the ethics committees will be undertaken. Details on monitoring, auditing and inspections will be captured in the study's monitoring plan.

6.0 PANEL OF EXPERTS

A panel of experts will provide advice to this study, they will include the following Collaborators: i) Dr. Hafsa Sentongo, Ag. Assistant Commissioner Mental Health and Control of Substance Use, Ministry of Health; ii) Dr Kenneth Kalani, Psychiatrist, Mental Health and Control of Substance Use, Ministry of Health; iii) Prof Vikram Patel, Department of Global Health and Social Medicine, Harvard Medical School; iv) Prof Ricardo Araya, Centre for Global Mental Health, Kings College; v) Dr. Giulia Greco, Global Health Economics Centre, LSHTM; vi) Prof Crick Lund, Centre for Global Mental Health, Kings College; vii) Prof. Valeria Mondelli, Maurice Wohl Clinical Neuroscience Institute, Kings College; viii) Dr Emmanuel Batibwe, Director Mubende Regional Referral Hospital.

7.0 REFERENCES

1. Centers for Disease Control and Prevention (CDC) (2021) Viral Haemorrhagic Fevers (VHFs). https://www.cdc.gov/vhf/virus-families/filoviridae.html
2. Centers for Disease Control and Prevention (CDC) (2019) 2014-2016 Ebola Outbreak in West Africa. https://www.cdc.gov/vhf/ebola/history/2014-2016-outbreak/index.html
3. World Health Organisation (WHO) (2022) Ebola Disease caused by Sudan virus - Uganda https://www.who.int/emergencies/disease-outbreak-news/item/2022-DON410
4. European Centre for Disease Prevention and Control (ECDPC) (2023) Ebola outbreak in Uganda, as of 11 January 2023. https://www.ecdc.europa.eu/en/news-events/ebola-outbreak-uganda

5. James PB, Wardle J, Steel A, Adams J (2019) Post-Ebola psychosocial experiences and coping mechanisms among Ebola survivors: a systematic review. *Trop. Med. Int. Health.* 24(6): 671–691.
6. Cénat JM, Felix N, Blais-Rochette C, Rousseau C, Bukaka J, Derivois D, Noorishad PG, Birangui JP (2020) Prevalence of mental health problems in populations affected by the Ebola virus disease: A systematic review and meta-analysis. *Psychiatry Res.* 289:113033.
7. Kaputu-Kalala-Malu C, Musalu EM, Walker T, Ntumba-Tshitenge O, Ahuka-Mundeke S (2021) PTSD, depression and anxiety in Ebola virus disease survivors in Beni town, Democratic Republic of the Congo. *BMC Psychiatry.* 21, 342.
8. Howlett PJ, Walder AR, Lisk DR, Fitzgerald F, Sevalie S, Lado M, N'jai A, Brown CS, Sahr F, Sesay F, Read JM, Steptoe PJ, Beare NAV, Dwivedi R, Solbrig M, Deen GF, Solomon T, Semple MG, Scott JT (2018) Case series of severe neurologic sequelae of Ebola Virus Disease during epidemic, Sierra Leone. *Emerg Infect Dis.* 24(8):1412-1421.
9. Kelly JD, Hoff NA, Spencer D, Musene K, Bramble MS, McIlwain D, Okitundu D, Porco TC, Rutherford GW, Glymour MM, Bjornson Z, Mukadi P, Okitolonda-Wemakoy E, Nolan GP, Muyembe-Tamfum JJ, Rimoin AW. (2019) Neurological, cognitive, and psychological findings among survivors of Ebola Virus Disease from the 1995 Ebola outbreak in Kikwit, Democratic Republic of Congo: A cross-sectional study. *Clin Infect Dis.* 68(8):1388-1393.
10. Lehmann M, Bruenahl CA, Löwe B, Addo MM, Schmiedel S, Lohse AW, Schramm C (2015) Ebola and psychological stress of health care professionals. *Emerg Infect Dis.* 21(5):913-4.
11. Druss BG, Hwang I, Petukhova M, Sampson NA, Wang PS, Kessler RC (2009) Impairment in role functioning in mental and chronic medical disorders in the United States: results from the National Comorbidity Survey Replication. *Mol. Psychiatry*. 14(7):728-37.
12. Sagui E, Janvier F, Baize S, Foissaud V, Koulibaly F, Savini H, et al. (2015) Severe Ebola virus infection with encephalopathy: evidence for direct virus involvement. *Clin Infect Dis.* 61:1627–8. 3.
13. Howlett P, Brown C, Helderman T, Brooks T, Lisk D, Deen G, et al. (2016) Ebola virus disease complicated by late-onset encephalitis and polyarthritis, Sierra Leone. *Emerg Infect Dis*. 22:150–2.
14. Kinyanda E, Kyohangirwe L, Mpango RS, Tusiime C, Ssebunnya J, Katumba K, Tenywa P, Mugisha J, Taasi G, Sentongo H, Akena D, Laurence Y, Muhwezi W, Weiss HA, Neuman M, Greco G, Knizek B, Levin J, Kaleebu P, Araya R, Ssembajjwe W, Patel V. (2021) Effectiveness and cost-effectiveness of integrating the management of depression into routine HIV Care in Uganda (the HIV + D trial): A protocol for a cluster-randomised trial. *Int J Ment Health Syst.* 15(1):45.
15. Patel V, Weiss HA, Chowdhary N, Naik S, Pednekar S, Chatterjee S, et al. (2010) Effectiveness of an intervention led by lay health counsellors for depressive and anxiety disorders in primary care in Goa, India (MANAS): a cluster randomised controlled trial. *Lancet.* 2010;376(9758):2086–95.

16. Wagner GJ, Ngo V, Glick P, Obuku EA, Musisi S, Akena D (2014) INtegration of DEPression Treatment into HIV Care in Uganda (INDEPTH-Uganda): study protocol for a randomized controlled trial. *Trials*. 15:248.
17. Kigozi FN, Ssebunnya J (2009) Integration of mental health into primary health care in Uganda: opportunities and challenges. *Ment Health Fam Med*. 6(1):37-42.
18. Galvin M, Byansi W. (2020) A systematic review of task shifting for mental health in sub-Saharan Africa. *Int. J. Ment. Health.* 49 (4): 336-360.
19. Patel V, Araya R, Chatterjee S, Chisholm D, Cohen A, De Silva M, Hosman C, McGuire H, Rojas G, van Ommeren M. (2007) Treatment and prevention of mental disorders in low-income and middle-income countries. *Lancet.* 370(9591):991-1005.
20. Ali BS, Rahbar MH, Naeem S, Gul A, Mubeen S, Iqbal A. (2003) The effectiveness of counseling on anxiety and depression by minimally trained counselors: a randomized controlled trial. *Am. J. Psychother*. 57(3):324-36.
21. Bell AC, D'Zurilla TJ. (2009) Problem-solving therapy for depression: a meta-analysis. *Clin Psychol Rev*. 29(4):348-53.
22. Cassano GB, Baldini Rossi N, Pini S. (2002) Psychopharmacology of anxiety disorders. *Dialogues Clin Neurosci*. 4(3):271-85.
23. Jakobsen JC, Katakam KK, Schou A. et al. (2017) Selective serotonin reuptake inhibitors versus placebo in patients with major depressive disorder. A systematic review with meta-analysis and Trial Sequential Analysis. *BMC Psychiatry*. 17, 58.
24. Zhen-Dong Huang Z-D, Yi-Fan Zhao Y-F, Li S, Gu H-Y, Lin L-L, Yang Z-Y, Niu Y-M, Zhang C, Luo J. (2020) Comparative efficacy and acceptability of pharmaceutical management for adults with Post-Traumatic Stress Disorder: A systematic review and meta-analysis. *Front. Pharmacol.* https://doi.org/10.3389/fphar.2020.00559
25. WHO. Geneva declaration on person centred care for chronic diseases. 2012. http://www.personcenteredmedicine.org/doc/2012_Geneva_Declaration_Final_19_May_2012.pdf
26. Beusenberg M, Orley JH, World Health Organization. (1994) Division of Mental Health. A User's guide to the Self-Reporting Questionnaire., World Health Organization. https://apps.who.int/iris/handle/10665/61113.
27. Nakimuli-Mpungu E, Mojtabai R, Alexandre PK, Katabira E, Musisi S, Nachega JB, Bass JK. (2012) Cross-cultural adaptation and validation of the self-reporting questionnaire among HIV+ individuals in a rural ART program in southern Uganda. *HIV AIDS (Auckl).* 4:51-60.
28. World Health Organization, CBM, World Vision International & UNICEF (2014) Psychological First Aid during Ebola virus disease outbreaks (provisional version). WHO, Geneva.
https://apps.who.int/iris/bitstream/handle/10665/131682/9789241548847_eng.pdf;jsessionid=E0C37B4BE8F9AEE0011A68BAB975E648?sequence=1

29. Hegel MT. (2011) Problem-Solving Treatment for Primary Care (PST-C): A treatment manual for depression, University of California, San Francisco. https://pstnetwork.ucsf.edu/sites/pstnetwork.ucsf.edu/files/documents/Pst-PC%20Manual.pdf
30. WHO. (2010) mhGAP intervention guide for mental, neurological and substance use disorders in nonspecialized health settings:Mental Health Gap Action Programme (mhGAP). http://apps.who.int/iris/bitstream/10665/44406/1/9789241548069_eng.pdf
31. Skivington K, Matthews L, Simpson SA, Craig P, Baird J, Blazeby JM, Boyd KA, Craig N, French DP, McIntosh E, Petticrew M, Rycroft-Malone J, Martin White M, Moore L. (2021) A new framework for developing and evaluating complex interventions: update of Medical Research Council guidance. *BMJ*. 374:n2061
32. Chowdhary N, Anand A, Dimidjian S, Shinde S, Weobong B, Balaji M, Hollon SD, Rahman A, Wilson GT, Verdeli H, Araya R, King M, Jordans MJD, Fairburn C, Kirkwood B, Patel V: The Healthy Activity Program lay counsellor delivered treatment for severe depression in India: systematic development and randomised evaluation. *British J Psychiatry*. 2016; 208, 381–388.
33. De Silva MJ, Breuer E, Lee L, Asher L, Chowdhary N, Lund C, Patel V: Theory of Change: a theory-driven approach to enhance the Medical Research Council's framework for complex interventions. *Trials*. 2014;15:267.
34. Vellakkal S, Patel V: (2015) Designing psychological treatments for scalability: The PREMIUM approach. *PLoS One*. 2015;10(7): e0134189.
35. Ssebunnya J, Mugisha J, Mpango R, Kyohangirwe L, Taasi G, Ssentongo H, et al. (2021). Using Theory of Change to inform the design of the HIV+D intervention for integrating the management of depression in routine HIV care in Uganda. *PLoS ONE*. 16(11): e0259425. https://doi.org/10.1371/journal.pone.0259425
36. Bell AC, D'Zurilla TJ. (2009) Problem-solving therapy for depression: a meta-analysis. *Clin Psychol Rev*. 29(4):348-53.
37. Zhang A, Park S, Sullivan JE, Jing S. (2018) The Effectiveness of Problem-Solving Therapy for Primary Care Patients' Depressive and/or Anxiety Disorders: A Systematic Review and Meta-Analysis. *J Am Board Fam Med*. 31(1):139-150.
38. Connolly SM, Vanchu-Orosco M, Warner J, Seidi PA, Edwards J, Boath E, Irgens AC. (2021) Mental health interventions by lay counsellors: a systematic review and meta-analysis. *Bull World Health Organ*. 99(8):572-582.
39. Singla DR, Weobong B, Nadkarni A, Chowdhary N, Shinde S, Anand A, Fairburn CG, Dimijdan S,Velleman R, Weiss H, Patel V. Improving the scalability of psychological treatments in developing countries: An evaluation of peer-led therapy quality assessment in Goa, India. *Behav Res Ther*. 2014; 60 (2014) 53e59.
40. Ede, V., et al. (2015) An examination of perceptions in integrated care practice. *Community Ment. Health J*. 51(8): 949-961.
41. Stahl ST, Albert SM, Dew MA, Anderson S, Karp JF, Gildengers AG, Butters MA, Reynolds CF 3rd (2017) Measuring Participant Effort in a Depression Prevention Trial: Who Engages in Problem-Solving Therapy? *Am J Geriatr Psychiatry*. 25(8):909-916.

42. Sheehan DV (1983) The Anxiety Disease. New York: Charles Scribner and Sons.
43. Findorff, M. J., et al. (2005). "Use of time studies for determining intervention costs." *Nurs. Res.* 54(4): 280-284.
44. Chapel JM, Wang G. (2019). "Understanding cost data collection tools to improve economic evaluations of health interventions." *Stroke Vasc. Neurol.* 4(4).
45. Drummond, M. F., et al. (2015). Methods for the economic evaluation of health care programmes, Oxford university press.
46. National Health Service (NHS) (2021) Side effects - Selective serotonin reuptake inhibitors (SSRIs). https://www.nhs.uk/mental-health/talking-therapies-medicine-treatments/medicines-and-psychiatry/ssri-antidepressants/side-effects/
47. Clarke A, Stein CR, Townsend ML (2008) Drug–Drug Interactions with HIV Antiretroviral Therapy. US Pharm.33(4):HS-3-HS-21. https://www.uspharmacist.com/article/drugdrug-interactions-with-hiv-antiretroviral-therapy
48. Heller, H.M., et al. (2017) Increased postpartum haemorrhage, the possible relation with serotonergic and other psychopharmacological drugs: a matched cohort study. *BMC Pregnancy and Childbirth.* 17(1):166.
49. British Journal of Obstetrics and Gynaecology: New studies show no long term effects of antidepressant use during pregnancy, but risk of significant blood loss during childbirth is increased. https://www.rcog.org.uk/en/news/bjog-release-use-of-antidepressants-during-pregnancy/, News 16 September 2015.
50. Kinyanda E, Weiss HA, Levin J, Nakasujja N, Birabwa H, Nakku J, Mpango R, Grosskurth H, Seedat S, Araya R, Patel V. (2017) Incidence and Persistence of Major Depressive Disorder Among People Living with HIV in Uganda. *AIDS Behav.* 21(6):1641-1654.
51. Kinyanda E, Hoskins S, Nakku J, Nawaz S, Patel V. (2011) Prevalence and risk factors of major depressive disorder in HIV/AIDS as seen in semi-urban Entebbe district, Uganda. *BMC Psychiatry.* 11:205.
52. Kinyanda E, Nakasujja N, Levin J, Birabwa H, Mpango R, Grosskurth H, Seedat S, Patel V. (2017) Major depressive disorder and suicidality in early HIV infection and its association with risk factors and negative outcomes as seen in semi-urban and rural Uganda. *J Affect Disord.* 212:117-127.
53. Musinguzi K, Obuku A, Nakasujja N, Birabwa H, Nakku J, Levin J, Kinyanda E. (20180 Association between major depressive disorder and pro-inflammatory cytokines and acute phase proteins among HIV-1 positive patients in Uganda. *BMC Immunol.* 19(1):1.
54. Kinyanda E, Waswa L, Baisley K, Maher D. (2011) Prevalence of severe mental distress and its correlates in a population-based study in rural south-west Uganda. *BMC Psychiatry*. 11, 97.
55. Kroenke K, Spitzer RL. (2002). The PHQ-9: a new depression diagnostic and severity measure. *Psychiatr. Ann.* 32(9), 509-515.
56. Akena D, Joska J, Obuku EA, Stein DJ. (2013). Sensitivity and specificity of clinician administered screening instruments in detecting depression among HIV-positive individuals in Uganda. *AIDS Care*. 25(10), 1245-1252.

57. Spitzer RL, Kroenke K, Williams JB, Löwe B. (2006). A brief measure for assessing generalized anxiety disorder: the GAD-7. *Arch. Intern. Med.* 166(10), 1092-1097.
58. Saunders JB, Aasland OG, Babor TF, De La Fuente JR, Grant, M. (1993). Development of the alcohol use disorders identification test (AUDIT): WHO collaborative project on early detection of persons with harmful alcohol consumption-II. *J. Addict.* 88(6), 791-804
59. Sheehan DV, Lecrubier Y, Sheehan KH, Amorim P, Janavs J, Weiller E, Hergueta T, Baker R, Dunbar GC. (1998) The Mini-International Neuropsychiatric Interview (M.I.N.I.): the development and validation of a structured diagnostic psychiatric interview for DSM-IV and ICD-10. *J Clin Psychiatry.* 59 Suppl 20:22-33;
60. Morin CM, Belleville G, Bélanger L, Ivers H. (2011). The Insomnia Severity Index: psychometric indicators to detect insomnia cases and evaluate treatment response. *Sleep.* 34(5), 601-608.
61. Gierk B, Kohlmann S, Kroenke K, et al. The Somatic Symptom Scale–8 (SSS-8): A Brief Measure of Somatic Symptom Burden. *JAMA Intern Med.* 2014;174(3):399–407.
62. Blevins CA, Weathers FW, Davis MT, Witte TK., Domino JL. (2015). The Posttraumatic Stress Disorder Checklist for DSM-5 (PCL-5): Development and initial psychometric evaluation. *Journal of Traumatic Stress.* 28(6), 489-498.
63. Antinori A, Arendt G, Becker JT, Brew BJ, Byrd DA, Cherner M, Clifford DB, Cinque P, Epstein LG, Goodkin K, et al. (2007) Updated research nosology for HIV-associated Neurocognitive disorders. *Neurology.* 69(18): 1789-1799.
64. Sacktor NC, Wong M, Nakasujja N, Skolasky RL, Selnes OA, Musisi S, Robertson K, McArthur JC, Ronald A, Katibira E (2005) The International HIV Dementia Scale: A new rapid screening test for HIV dementia. *AIDS.* 19:1367-1374.
65. Selnes OA, Jacobson L, Machado AM, Becker JT, Wesch J, Miller EN, Visscher B, McArthur JC (1991) Normative data for a brief neuropsychological screening battery. Multicenter AIDS Cohort Study. *Percept Mot Skills.* 73(2):539-50.

66. Musaazi J, Namageyo-Funa A, Carter VM, Carter RJ, Lamorde M, Apondi R, Bakyaita T, Boore AL, Brown VR, Homsy J, Kigozi J, Koyuncu A, Nabaggala MS, Nakate V, Nkurunziza E, Stowell DF, Walwema R, Olowo A, Jalloh MF. (2020) Evaluation of Community Perceptions and Prevention Practices Related to Ebola Virus as Part of Outbreak Preparedness in Uganda. *Glob. health, Sci. Pract.* June 2022, 10(3):e2100661.
67. Kerkhof A, Bernasco W, Bille-Brahe U, Platt S, Schmidtke A. WHO/EURO multicentre study on parasuicide: European parasuicide study interview schedule (EPSIS). Leiden: Department of Clinical Health and Personality Psychology, Leiden University; 1989.
68. Kinyanda, E., Hjelmeland, H., & Musisi, S. (2005). Negative life events associated with deliberate self-harm in an African population in Uganda. *Crisis.* 26(1), 4.
69. Carver, C. S. (1997). You want to measure coping but your protocol'too long: Consider the brief cope. *IJBM.* 4(1), 92-100.

70. Connor, K. M., & Davidson, J. R. (2003). Development of a new resilience scale: The Connor-Davidson resilience scale (CD-RISC). *Depress Anxiety.* 18(2), 76-82.
71. Dahlem NW, Zimet GD, Walker RR. (1991) The multidimensional scale of perceived social support: a confirmation study. *J Clin Psychol.* 47(6):756–61.
72. Wauquier N, Becquart P, Padilla C, Baize S, Leroy EM. (2010) Human fatal zaire ebola virus infection is associated with an aberrant innate immunity and with massive lymphocyte apoptosis. *PLoS Negl Trop Dis.*4(10):e837.
73. Jacobs, M., et al. (2016) Late Ebola virus relapse causing meningoencephalitis: a case report. *Lancet.* 388(10043): 498-503.
74. Ogunmokun G, Dewanjee S, Chakraborty P, Valupadas C, Chaudhary A, Kolli V, Anand U, Vallamkondu J, Goel P, Paluru HPR, Gill KD, Reddy PH, De Feo V, Kandimalla R. (2021) The Potential Role of Cytokines and Growth Factors in the Pathogenesis of Alzheimer's Disease. *Cells.* 10(10):2790.
75. Ramesh G, MacLean AG, Philipp MT. (2013) Cytokines and chemokines at the crossroads of neuroinflammation, neurodegeneration, and neuropathic pain. *Mediators Inflamm.* :480739.
76. Bhattacharya A, Ashouri R, Fangman M, Mazur A, Garett T, Doré S. (2021) Soluble Receptors Affecting Stroke Outcomes: Potential Biomarkers and Therapeutic Tools. *Int J Mol Sci.* 22(3):1108.
77. Bio-RAD (2023) Luminex xMAP Technology. https://www.bio-rad.com/featured/en/luminex-xmap-technology.html#:~:text=Luminex%20Overview,spectral%20addresses%20(color%20codes).
78. Hackman, J. R., & Oldham, G. R. (2005). How job characteristics theory happened. In. K. Smith, & M. Hitt. Great minds in management: The process of theory development, 151-170.
79. Gea-Caballero V, Juárez-Vela R, Díaz-Herrera MÁ, Mármol-López MI, Blazquez RA, Martínez-Riera JR. (2019). Development of a short questionnaire based on the Practice Environment Scale-Nursing Work Index in primary health care. *Peer J.* 7: e7369.

8.0 APPENDICES

Appendix I: Ebola+D health systems quantitative and qualitative questionnaires

Appendix II: Ebola+D cohort sub-study quantitative questionnaires

Appendix III: Consent documents

Appendix IV: Standard Operating Procedures (SOPs)
